# The evolutionary pathway from a biologically inactive polypeptide sequence to a folded, active structural mimic of DNA

**DOI:** 10.1093/nar/gkw234

**Published:** 2016-04-19

**Authors:** Nisha Kanwar, Gareth A. Roberts, Laurie P. Cooper, Augoustinos S. Stephanou, David T.F. Dryden

**Affiliations:** EaStCHEM School of Chemistry, University of Edinburgh, The King's Buildings, Edinburgh EH9 3FJ, UK

## Abstract

The protein Ocr (overcome classical restriction) from bacteriophage T7 acts as a mimic of DNA and inhibits all Type I restriction/modification (RM) enzymes. Ocr is a homodimer of 116 amino acids and adopts an elongated structure that resembles the shape of a bent 24 bp DNA molecule. Each monomer includes 34 acidic residues and only six basic residues. We have delineated the mimicry of Ocr by focusing on the electrostatic contribution of its negatively charged amino acids using directed evolution of a synthetic form of Ocr, termed pocr, in which all of the 34 acidic residues were substituted for a neutral amino acid. *In vivo* analyses confirmed that pocr did not display any antirestriction activity. Here, we have subjected the gene encoding pocr to several rounds of directed evolution in which codons for the corresponding acidic residues found in Ocr were specifically re-introduced. An *in vivo* selection assay was used to detect antirestriction activity after each round of mutation. Our results demonstrate the variation in importance of the acidic residues in regions of Ocr corresponding to different parts of the DNA target which it is mimicking and for the avoidance of deleterious effects on the growth of the host.

## INTRODUCTION

Nature is replete with examples of mimicry, both at the macro and molecular level. Phage- or plasmid-encoded antirestriction proteins that act as structural mimics of DNA alleviate the effects of host restriction during invasion of the host by phage or plasmid DNA (1–6). To date, the bacteriophage T7 protein Ocr is probably the best characterised structural mimic of double stranded DNA (7–18). Ocr is able to competitively inhibit Type I restriction modification (RM) systems found in eubacteria.

Type I RM systems are complex oligomeric multifunctional enzymes that can mediate both restriction and modification of a DNA substrate (19–22). Recent analyses have confirmed that Type I RM systems are widely found in diverse eubacteria and provide a formidable barrier to the exchange of DNA by horizontal gene transfer or phage infection (23). The Type I RM system operates by modifying the host DNA *via* methylation of specific bases at an asymmetric bipartite recognition sequence containing a central non-specific spacer region which marks it as being ‘self’ (24). For example, the EcoKI Type I RM enzyme recognises 5′-AACNNNNNNGTGC-3′ within a larger footprint and determines the methylation status of the adenine at the underlined bases (25,26). DNA entering the cell (e.g. during phage infection) that lacks the appropriate pattern of modification will be recognised as ‘foreign’ and rendered a target for degradation by the endonuclease component of the Type I RM system. The Type I RM enzyme comprises three different subunits, S, M and R, encoded by three discrete genes, *hsdS, hsdM* and *hsdR*, respectively. Depending on the methylation status of the DNA substrate, the RM complex functions as either a restriction endonuclease or a methyltransferase. Specifically, the M_2_S_1_ complex acts as a sequence-specific methyltransferase on hemimethylated DNA, whereas the complete enzyme, R_2_M_2_S_1_, displays endonuclease activity on unmethylated DNA (27–29). The S subunit identifies the DNA recognition sequence, the two M subunits contain the methyltransferase activity and the two R subunits house the ATP-dependent translocase and endonuclease activity. *S*-adenosylmethionine (SAM) is the methyl donor for the methyltransferase activity, whilst the endonuclease activity requires ATP, SAM and Mg^2+^.

**Figure 1.**
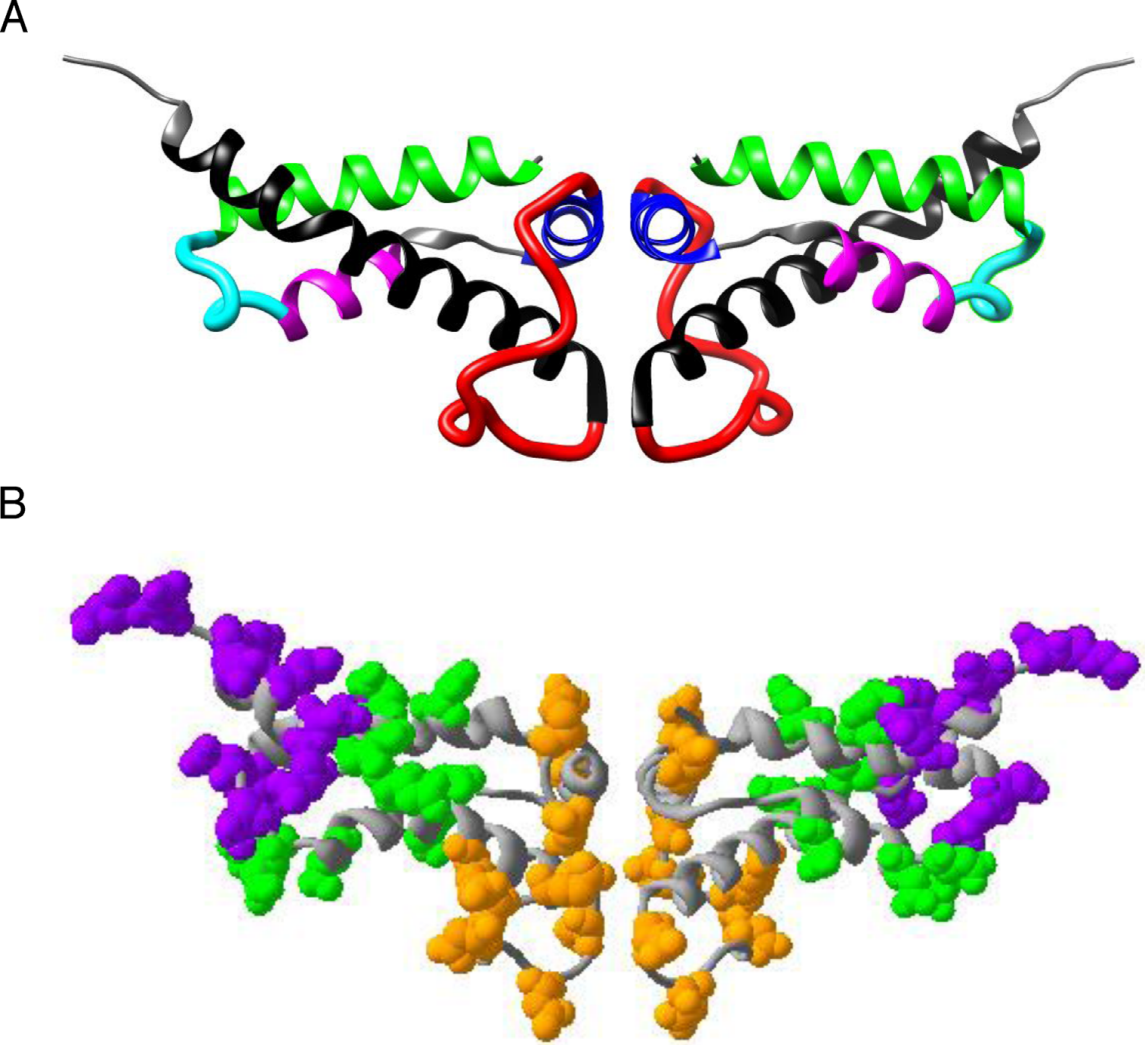
(**A**) The structure of the dimeric Ocr protein (PDB 1S7Z) showing the alpha helices (A in green, B in magenta, C in blue and D in black), Loop 1 (cyan) and Loop 2 (red). The N termini of the monomers are at the dimer interface and the C termini at the outer edges of the dimer. (**B**) The structure of the Ocr dimer highlighting the acidic amino acids overlapping with different regions of the DNA target recognised by Type I RM enzymes. The orange region overlaps with the central non-specific region, green overlaps with the two specific parts of the target sequence and purple overlaps with DNA flanking the target sequence.

Bacteriophage T7 gene 0.3, which encodes Ocr, is the first phage gene to be expressed during the infection of *Escherichia coli* (1,2). The newly produced Ocr then binds, almost irreversibly, to the Type I RM system and protects the unmodified phage DNA as it enters the host cell by inhibiting the endonuclease activity (7). The structure of Ocr reveals the protein to be a remarkable mimic of bent B-form DNA approximately 24-bp in length (8,9,11). Ocr is a homodimer of a polypeptide of 116 amino acids which includes an overabundance of acidic residues (30,31). The structure features several alpha helices and loops (Figure 1a). The overall shape of the protein resembles that of a bent DNA duplex (∼34° in the helical axis) and the numerous surface-exposed carboxylic acid groups are thought to mimic the phosphate backbone of the DNA molecule (10,11). Furthermore, the acidic sidechains can be divided into regions corresponding to the regions of DNA target sequence recognised by the Type I RM enzyme (15), namely a central region corresponding to the non-specific central part of the target sequence (coloured orange in Figure 1b) flanked by two regions corresponding to the sequence-specific parts of the target sequence (coloured green in Figure 1b) and outermost are two regions corresponding to non-specific DNA making up the remainder of the large footprint of the RM enzyme (coloured purple in Figure 1b). The bent shape of Ocr is believed to simulate the bend induced in the DNA substrate upon binding to the Type I RM enzyme (32). In this way, the ‘pre-bent’ Ocr molecule is energetically more favoured as a binding partner to the Type I RM enzyme than an unbent DNA molecule. Hence, the overall binding affinity of the Type I RM system EcoKI for Ocr is approximately 50-fold greater than for DNA (7,8,10,16–18). Thus, a combination of the shape component together with the electrostatic mimicry facilitates the efficient inhibition of the Type I RM system. Although Ocr is a highly effective mimic of DNA, the mimicry is not sequence specific (3,7,11). Hence, Ocr is able to inhibit all Type I RM systems, rather than one particular enzyme.

We previously investigated the electrostatic component of the mimicry displayed by Ocr. Initially, we attempted to analyse the electrostatic contribution of the mimicry by chemically modifying Ocr to neutralise the surface exposed carboxylates whilst simultaneously retaining the overall structure of the protein (13). We found that Ocr requires a minimum of ∼16 acidic residues per monomer to act as an effective antirestriction protein. However, the chemical modification procedure is essentially a random process in which surface exposed carboxylates are subject to neutralisation. As such, the positional context of acidic residues within Ocr that are particularly important for its interaction with M.EcoKI (the M_2_S_1_ core of the RM enzyme) could not be established using this approach.

Following the chemical modification experiments, we substituted acidic residues for neutral amino acids in specific structural regions of Ocr using site-directed mutagenesis (14,15). Mutagenesis of up to 17 amino acids per monomer resulted in no significant alteration in the protein fold as based on CD analysis and structural stability studies. Examination of these mutant proteins, both *in vitro* and *in vivo*, indicated that acidic residues located in the central loop region (loop 2) of Ocr are particularly important in terms of its interaction with the archetypal Type I RM system EcoKI (Figure 1). However, these mutagenesis studies were obviously limited to analysing a relatively small number of mutated forms of Ocr. We therefore sought an independent method to identify acidic residues of Ocr that are principally involved in its DNA mimicry.

During the course of the mutagenesis studies (15) we generated a mutant form of Ocr, termed pocr, in which all 34 acidic residues had been substituted for the corresponding isogenic neutral amino acid (i.e. Asp to Asn and Glu to Gln). The pocr gene was then engineered for heterologous expression in *Escherichia coli*. However, despite numerous attempts, we were unable to detect the accumulation of recombinant pocr, suggesting the protein is unable to properly fold and/or is inherently unstable. Moreover, phage restriction assays showed pocr to display no detectable antirestriction activity. These finding were unsurprising given that pocr comprises a mutation frequency of almost 30% of the residues found in Ocr. Nonetheless, we reasoned that the gene encoding pocr could act as an appropriate starting point for a directed evolution study aimed at generating an Ocr-like protein with antirestriction activity.

The directed evolution study described in this paper was designed to introduce codons for acidic amino acids into appropriate sites in the pocr gene in order to specifically substitute neutral residues found in pocr for acidic residues found in Ocr (i.e. Asn to Asp; Gln to Glu as required). Conditions were chosen to mutate four or five codons per round of mutation in a random unbiased manner. This process can be viewed as a progressive substitution of neutral residues in pocr, which displays no antirestriction activity, towards the generation of Ocr, which obviously possesses antirestriction activity. By subjecting libraries of such pocr mutants to a powerful plate-based selection procedure after each round of mutagenesis we hoped to identify an Ocr-like protein with antirestriction activity containing a much reduced number of acidic residues.

## MATERIALS AND METHODS

### Materials

Unless otherwise stated all the chemicals used in this study were purchased from Sigma-Aldrich (St Louis, MO, USA). pBRsk1 (33) is an engineered version of pBR322 (4361 bp) in which one of the two EcoKI sites (4024–4036) has been removed by site-directed mutagenesis. Plasmid pET24a was purchased from Novagen (Madison, WI, USA) and pTrc99A was a gift from Dr Angela Dawson (School of Physics and Astronomy, University of Edinburgh). A synthetic version of the gene encoding Ocr in which all the codons for acidic acid residues had been mutated to codons for the corresponding isogenic neutral residues (Asp and Glu to Asn and Gln, respectively), termed pocr, was created by GeneArt (Regensberg, Germany). Codon usage was optimised for *E. coli* (Supplementary Figure S1). *E. coli* XL1 Blue supercompetent cells were obtained from Stratagene (La Jolla, CA, USA) and *E. coli* BL21(DE3) was from Invitrogen Life Technologies (Carlsbad, CA, USA). *E. coli* NM1261 (r^−^m^−^; no RM system) and *E. coli* NM1049 (r^+^m^+^; EcoKI RM system) were a kind gift of Professor Noreen Murray (School of Biology, University of Edinburgh, UK). These two NM strains were converted to DE3 lysogens as described in (6). *E. coli* NM1041 (*E. coli* MG1655 r^+^m^+^; EcoKI RM system clpX::kan) and *E. coli* NM1057 (*E. coli* MG1655 rm^+^; no RM system, ΔhsdR_EcoKI_, clpX::kan) were kind gifts from Dr Angela Dawson. Virulent unmodified bacteriophage lambda λ_v.o_, or EcoKI-modified lambda λ_v.k_ were kindly provided by Professor Noreen Murray. Isoelectric point, pI, values were calculated using the ExPaSy compute pI/Mw tool in the bioinformatics research portal.

### Library construction

Standard DNA manipulation procedures were used throughout (34). Directed evolution of pocr was performed using a modified version of the ISOR protocol described by Herman and Tawfik (35,36). The libraries were constructed with desalted oligonucleotides obtained from Invitrogen (Paisley, UK). Initially, the pocr gene ligated into pET24b was amplified with oligonucleotides that primed from within the vector to give a PCR product of 500 bp. The resulting flanking regions of the PCR product covering part of the vector sequence facilitated manipulation of the relatively short pocr gene and assisted in the PCR reassembly step. DNase I digestion of the PCR product was conducted and the mixture was resolved on a 2% agarose gel before extracting fragments of <100 bp in length. Mutagenic oligonucleotides were designed to mutate codons for neutral amino acids in pocr to the corresponding codons for acidic residues found in Ocr (i.e. Asn to Asp; Gln to Glu). To circumvent problems associated with overlapping mutagenic oligonucleotides, the ISOR procedure was conducted in four separate reactions targeting four discrete sets of non-contiguous codons. In order to achieve an average mutation frequency of four or five mutated codons per round of ISOR, the total concentration of mutagenic oligonucleotides was fixed at 200 nM. Equimolar amounts of each oligonucleotide were used throughout. The sequences of the four pools of mutagenic oligonucleotide primers used in this study are shown in Supplementary Table S1. The four pools of DNA obtained by ISOR were then shuffled by DNase I digestion and PCR reassembly to generate the library for screening. Primers Nfor, 5′-GTATCACCATGGCTATGTCTAACATG-3′ and Nrev 5′-GATCCCCCGTCAAGCTTA-3′ were designed to amplify the assembled pocr library and introduce NcoI and HindIII restriction sites (underlined) in a nested PCR reaction to facilitate insertion of the DNA into pTrc99A.

In all, three rounds of directed evolution were performed. For the final round of ISOR, degenerate oligonucleotides incorporating more than one mutation were also used. This step was necessary because the shuffling procedure was inefficient and led to under-representation in the number of consecutively mutated codons. Consequently, a bias emerged in the library after the second round that favoured mutations which were well separated from each another. Unfortunately, selecting shorter DNA fragments after DNase I digestion in the shuffling procedure made the PCR reassembly inefficient. To circumvent such problems, the third round of mutation included degenerate oligonucleotides covering successive mutation sites (Supplementary Table S2). Using this strategy, a degree of randomness in the construction of the library was maintained. There was, however, a marked reduction in the fidelity of library construction in terms of the number of unwanted alterations during this round of directed evolution. Specifically, we noticed that ∼10% of the library after the third round of directed evolution was made up of pocr constructs containing either deletions or frameshifts. It is unclear why the introduction of oligonucleotides containing more than one mutation should result in this reduced fidelity, although it may be related to mispriming events. Nonetheless, we judged that the library was sufficiently diverse to proceed with the selection assay.

### Selection assay procedure

Each plasmid library was initially used to transform XL-Gold Supercompetent cells at high efficiency by following the manufacturer's instructions (Stratagene). The complete transformation mix was plated to ensure the diversity of the respective library was fully represented. The resulting colonies from all of the plates were pooled using 3 ml of LB per plate and the plasmid DNA library was prepared using a Qiagen Maxiprep kit (Qiagen, Hilden, Germany). Each library was quantified using a ND1000 nanodrop spectrophotometer (Thermo Scientific, Waltham, MA, USA). The plasmid library (1 or 4 ng) was then used to transform *E. coli* NM1041 cells. Quenching experiments were carried out by plating known amounts of *E. coli* NM1041 cells with increasing concentrations of 2-aminopurine (2AP). A 2AP concentration of 80 μg/ml was found to be effective for selecting 2000 colonies of *E. coli* NM1041 cells per plate (90 mm diameter Petri dish). Therefore 100 ng of plasmid DNA was used to transform 200 μl of *E. coli* NM1041 chemically competent cells and 100 μl aliquot (∼2000 colonies) of the transformation mix was spread on each plate. To analyse 1.2 × 10^6^ clones for each library, the selection process was split into three batches. For each batch, 20 separate transformations of *E. coli* NM1041 with the plasmid DNA library were plated onto LB-agar plates supplemented with 2AP (80 μg/ml) and incubated overnight at 37°C. The plates were then then left at room temperature for three days. Positive clones from the initial selection screen were re-streaked onto 2AP-containing plates (80 μg/ml) for a second screen. After overnight incubation at 37°C, any positive clones were picked and plasmid DNA prepared. The plasmid was used to transform *E. coli* NM1041 which were selected for 2AP resistance as before. Mutated versions of pocr isolated from the 2AP-resistant cells were subsequently sequenced (Genepool, Edinburgh University, Edinburgh, UK).

### *In vivo* phage restriction and modification assay

The activity of the selected pocr mutants was assessed using an *in vivo* assay with virulent bacteriophage *λ*_v.o_ or *λ*_v.k_ as described previously (5).

### Protein purification

Recombinant protein was obtained using *E. coli* BL21(DE3) as host and purified as described previously (13,14). M.EcoKI and EcoKI were prepared as previously described (27,37). All pocr and Ocr protein concentrations refer to the dimeric form of the protein.

### Glutaraldehyde crosslinking

Chemical crosslinking was used to assess whether each of the pocr mutant proteins exist as a homodimer in solution. A 25 μg aliquot of each protein was processed using the method described in (15).

### Circular dichroism for assessment of pocr folding

Far UV (190–260 nm) circular dichroism (CD) analysis was performed on a Jasco model J-180 spectropolorimeter (Jasco Corporation, Tokyo, Japan) at 25°C in 10 mM sodium phosphate buffer pH 8.0, containing 50 mM NaF and 7 mM mercaptoethanol at a protein concentration of 30 μM using a 1 mm pathlength cell. Spectra were corrected for buffer contribution. Each spectrum was an accumulation of four individual scans.

### Thermal denaturation

The transition melting temperature *T*_m_ of the protein was measured by a thermal denaturation assay. The assay measures the fluorescence emitted by the dye SYPRO orange (Invitrogen, Burlington, Canada), which is strongly quenched in an aqueous environment. The SYPRO orange (5000-fold dilution of stock provided by Invitrogen) was added to the protein samples in 10 mM HEPES buffer, pH 8.0 and the temperature was controlled using a qPCR machine.

### Isothermal titration calorimetry

The binding of selected pocr mutants to M.EcoKI was assessed by isothermal titration calorimetry (ITC) as described in (15) but with the substitution of 20 mM HEPES buffer, pH 7.0.

### *In vitro* DNA cleavage assay

The ability of each pocr mutant to inhibit the endonuclease activity of EcoKI was assessed *in vitro* as described in (15) with the substitution of 10 mM HEPES buffer, pH 7.0 and the use of 5:1 and 10:1 ratios of pocr mutant to EcoKI and a reaction time of 10 minutes.

### Microscopy and bacterial growth curve experiments

These experiments were performed as described in (15). Growth curves were determined using *hsdR*^−^ and *hsdR*^+^ strains *E. coli* NM1261 and *E. coli* NM1041.

## RESULTS

### Directed evolution of pocr

We aimed to progressively mutate all 34 targeted codons of the pocr gene in an unbiased manner. To achieve this objective, we employed a modified version of the ISOR methodology (35,36) in which codons for acidic residues replaced codons for neutral amino acids in a stepwise fashion to generate a series of libraries. ISOR is a DNA shuffling technique, which generates mutations by assembly of the gene of interest using oligonucleotides containing the desired mutation at the appropriate position. In general, conditions were chosen that favoured the introduction of an average of four or five mutated codons for each round of directed evolution.

To the best of our knowledge, ISOR has not previously been used to mutate small genes, such as pocr, where the targeted sites for mutation are often in close proximity to one another. As far as possible, mutagenic oligonucleotides were designed with a similar melting temperature (Tm) and GC content in order to reduce bias during the assembly process. For each round of mutation, separate mutagenesis reactions were carried out in which four different sets of codons were independently targeted (Supplementary Figure S2 and Supplementary Table S1). Using this strategy, codons in close proximity to one another could be mutated without the mutagenic oligonucleotides overlapping. Herman and Tawfik (35) reported that the mutation frequency in this procedure could be enhanced by increasing the concentration of mutagenic oligonucleotides. In our experiments, a desired mutation rate of approx. four to six mutations per round of mutagenesis was achieved using an oligonucleotide concentration of 200 nM (oligonucleotide concentrations ≥360 nM inhibited the reaction). To reduce the possibility of introducing bias, an equimolar amount of each oligonucleotide was used in all cases.

The mutations introduced in each of the four separate reactions produced four sub-libraries, which were subsequently mixed and shuffled by DNase I digestion followed by PCR reassembly. Analysis of the resulting library determined whether the shuffling process was efficient. A total of 30 clones were randomly picked from the library and plasmid DNA was prepared from each and sequenced in order to monitor the average number of introduced mutations. In this way, we were able to assess the overall fidelity of the procedure. Our analysis suggested an average of five mutations were generated during the first round of mutation with no clones containing undesired changes (e.g. non-targeted mutations, frameshifts, truncations, duplications, etc.). A small proportion (∼1–3%) of the library was made up of plasmid with no insert (empty vector). This was not deemed to be a problem because empty vector was lost from the process during the subsequent amplification of the pocr insert prior to the next round of mutagenesis. The shuffled library was then subjected to the first round of the selection procedure. A plate-based assay was used to screen approximately 1.2 × 10^6^ clones from the first library. No clones displaying antirestriction activity were detected using this initial selection process. Therefore, total DNA from the first library was pooled and subjected to further rounds of ISOR mutagenesis followed by selection.

In all, three rounds of mutation using the ISOR procedure were required before the appearance of clones with detectable antirestriction activity. Sequence analysis of the second and third round libraries showed the average number of mutated codons was 6–13 and 8–17, respectively. Approximately 1.2 × 10^6^ variants from each library were screened after each successive round of directed evolution.

### Selection process

For screening, the library was ligated into the NcoI and HindIII sites of the pTrc99A vector and the ligation mix was used to transform *E. coli* NM1041. This strain has a functioning Type I RM system (EcoKI) but lacks the ClpXP protease, which destroys the R subunit of EcoKI when DNA damaging agents are present. As such, the *E. coli* NM1041 strain is exquisitely sensitive to the presence of 2AP, which is both an adenine analogue and a powerful mutagen. When 2AP is present, unmodified sites occur on the host chromosome and EcoKI cleaves the chromosome. As such, *E. coli* NM1041 plated on solid medium containing 2AP at a concentration of >20 μg/ml will not survive. However, the cells are able to grow if the strain harbours an inhibitor of the restriction endonuclease, such as Ocr.

Thus, the selection process involved plating out the *E. coli* NM1041 transformed with the library of pocr mutants onto solid LB medium supplemented with 50 μg/ml carbenicillin and 80 μg/ml 2AP. The presence of a pocr mutant that can act as an effective DNA mimic will inhibit the restriction endonuclease and enable survival. Any clones that grew were picked and then subjected to a second round of screening using 2AP.

No positive clones were isolated from the first or second rounds of directed evolution. However, the third round of screening generated seven positive clones (M1.1, M1.4, M1.7, M1.9, M2.1, M2.8 and M2.11). The clones were verified by retransformation of *E. coli* NM1041 with the isolated plasmids and then selection using plates supplemented with 2AP. Plasmid DNA was isolated from each of the seven positive clones and the insert was sequenced. Clones M1.7 and M1.9 were found to be identical to each other as were clones M1.4, M2.1 and M2.11. Thus, our analysis revealed four of the seven clones contained unique sequences. In all four cases, mutations of pocr were confined to the targeted codons with no other unanticipated changes. Moreover, each of the introduced point mutations resulted in a Glu for Gln or Asp for Asn substitution and the total number of mutations in the four isolated clones were 16, 16, 14 and 17 for M2.1, M1.1, M1.9 and M2.8, respectively.

### *In vivo* phage restriction assay

The activity of the selected pocr mutants was assessed *in vivo* using a restriction assay (Table 1). Infection was carried out with λ_v.o_ of two different *E. coli* strains, transformed with pTrc99A or the plasmids expressing the pocr mutants or Ocr as a control. *E. coli* NM1049(DE3) contains the EcoKI RM system (r^+^m^+^) while *E. coli* NM1261(DE3) lacks the RM system (r^−^m^−^). The efficiency of plating (eop) of λ_v.o_ on *E. coli* NM1049(DE3) pTrc99A compared to *E. coli* NM1261(DE3) pTrc99A was 5 × 10^−5^ indicating that EcoKI was active. Comparing the two strains when transformed with the plasmid expressing the wild-type Ocr gave a similar number of plaques and an eop of 4, demonstrating that Ocr had inhibited the Type I RM system as anticipated. Assaying the four selected pocr mutants in the same way showed that all were fully active with an eop ranging from 1 to 3. Control experiments using *λ*_v.k_ showed there was no cut back in the number of plaques on the restrictive versus the non-restrictive strain.

**Table 1.** *In vivo* antirestriction and antimodification activity of Ocr and selected pocr mutant proteins

Plasmid	Efficiency of plating on strain transformed with plasmid	Efficiency of plating after recovery from modifying strain transformed with plasmid
Vector pTrc99A	5×10^−5^	1
Ocr	4	1.4×10^−4^
M2.8	2	2.7×10^−4^
M2.1	2	1.7×10^−4^
M1.1	3	2.1×10^−4^
M1.9	1	1.7×10^−4^

The titre of phage λ_v.o_ per millilitre was determined in *E. coli* NM1261(DE3) (r^−^m^−^) and *E. coli* NM1049(DE3) (r^+^m^+^) transformed with either the vector alone (pTrc99A) or plasmid expressing Ocr or pocr mutant (M2.8, M2.1, M1.1 or M1.9). The ratio of phage per ml in *E. coli* NM1049(DE3) to phage per ml in *E. coli* NM1261(DE3) was calculated to obtain the efficiency of plating (eop). For the antimodification assays, unmodified phage λ_v.o_ grown on *E. coli* NM1057 (r^−^m^+^, clpX^−^) in the presence or absence of Ocr or pocr mutant were recovered and a phage stock generated. The degree of antimodification was assessed by determining the eop of each phage stock on a restriction-proficient strain (*E. coli* NM1041 (r^+^m^+^, clpX^−^)), relative to a non-restricting strain (*E. coli* NM1057 (r^−^m^+^, clpX^−^)). Errors in the number of plaques where ±20% from full plate assays performed in triplicate.

### *In vivo* phage modification assay

To assess the methylation activity of each of the mutants, a phage modification assay was carried out. λ_v.o_ was used to infect the modifying host *E. coli* NM1057 (r^−^m^+^) transformed with either pTrc99A or with pTrc99A expressing Ocr or the selected pocr mutants. *E. coli* NM1057 expresses M.EcoKI so phages recovered from a single plaque after infection should have been methylated by the action of M.EcoKI unless the pocr mutants successfully inhibited the methyltransferase activity. Activity was assessed by determining the eop of the phages recovered from a single plaque on *E. coli* NM1057. The eop was determined on the restrictive host *E. coli* NM1041 (r^+^m^+^, clpX^−^) relative to the modifying host *E. coli* NM1057 (r^−^m^+^, clpX^−^). If the pocr mutant displays antimodification activity similar to Ocr then the titre of recovered phages per ml after plating λ_v.o_ on *E. coli* NM1041 would be lower than the titre on *E. coli* NM1057. The results showed that wild-type Ocr and all of the selected pocr mutants were active in preventing DNA modification by M.EcoKI as their eop was 1 × 10^−4^ to 3 × 10^−4^ (Table 1).

### Protein purification

All four of the unique pocr clones selected from the third round of mutation gave similar levels of expression, equivalent to that observed for wild-type Ocr and were purified to apparent homogeneity using the same protocol employed for the purification of wild-type Ocr (data not shown, 13,14).

### Evaluation of the protein fold of the pocr mutants

Far UV CD analysis of each of the four pocr mutants was performed along with Ocr for comparison (Supplementary Figure S3). The spectra were analysed using the Dichroweb online secondary structure deconvolution program (38). The CDSSTR method gave a α-helical content of ∼60% for Ocr, which is in good agreement with the crystal structure. Similar values (±2%) were obtained for each of the pocr mutants, indicating that the absence of approximately half of the acidic residues present on Ocr has no significant effect on its secondary structure. Thus, it appears that many of the acidic residues on the wild-type Ocr protein have no substantial bearing on its overall structural integrity. Wild-type Ocr is known to exist as a homodimer in solution. Glutaraldehyde crosslinking confirmed that the selected pocr mutants also exist as dimers (Supplementary Figure S4). The thermal stability of the pocr mutants was assessed using a fluorescence-based denaturation assay. Wild-type Ocr, M1.1, M2.1 and M2.8 all gave a similar *T*_m_ value of 54–57°C under the conditions used in our assay. Intriguingly, however, mutant M1.9 displayed a *T*_m_ of 67°C, indicating an enhanced thermal stability (Supplementary Figure S5). The curious enhancement in thermal stability of M1.9 may be related to an increase in the distance between discrete carboxylates on the protein surface, which reduces electrostatic repulsion. Indeed, the closeness of the carboxylates in Ocr is approximately equal to the Bjerrum length, leading to electrostatic repulsion energies of the order *k*_B_*T* (39).

### Isothermal titration calorimetry

ITC was used to analyse the interaction between M.EcoKI and each of the four unique pocr proteins. Wild-type Ocr was used as a positive control. Each injection peak was integrated and the heat of dilution subtracted. A plot of the heat of interaction *versus* the molar ratio of Ocr dimer to M.EcoKI was then generated (Supplementary Figure S6). As found previously (10,13), the interaction between wild-type Ocr and M.EcoKI was highly exothermic and exhibited essentially stoichiometric binding (one Ocr dimer per M.EcoKI) within the concentration range used in these experiments (Supplementary Figure S6). However, the binding of Ocr to M.EcoKI was too strong to allow determination of the dissociation constant, which has been estimated as ∼50 pM using other methods (10). For the pocr mutants the enthalpy of interaction with M.EcoKI varied with each mutant. The pocr mutant proteins M2.1, M2.8 and M1.1 displayed a highly exothermic interaction with M.EcoKI similar to that observed for wild-type Ocr under the same experimental conditions. By contrast, the interaction of M1.9 with M.EcoKI showed a markedly reduced enthalpy of interaction (Supplementary Figure S6).

### Nuclease assay

**Figure 2.**
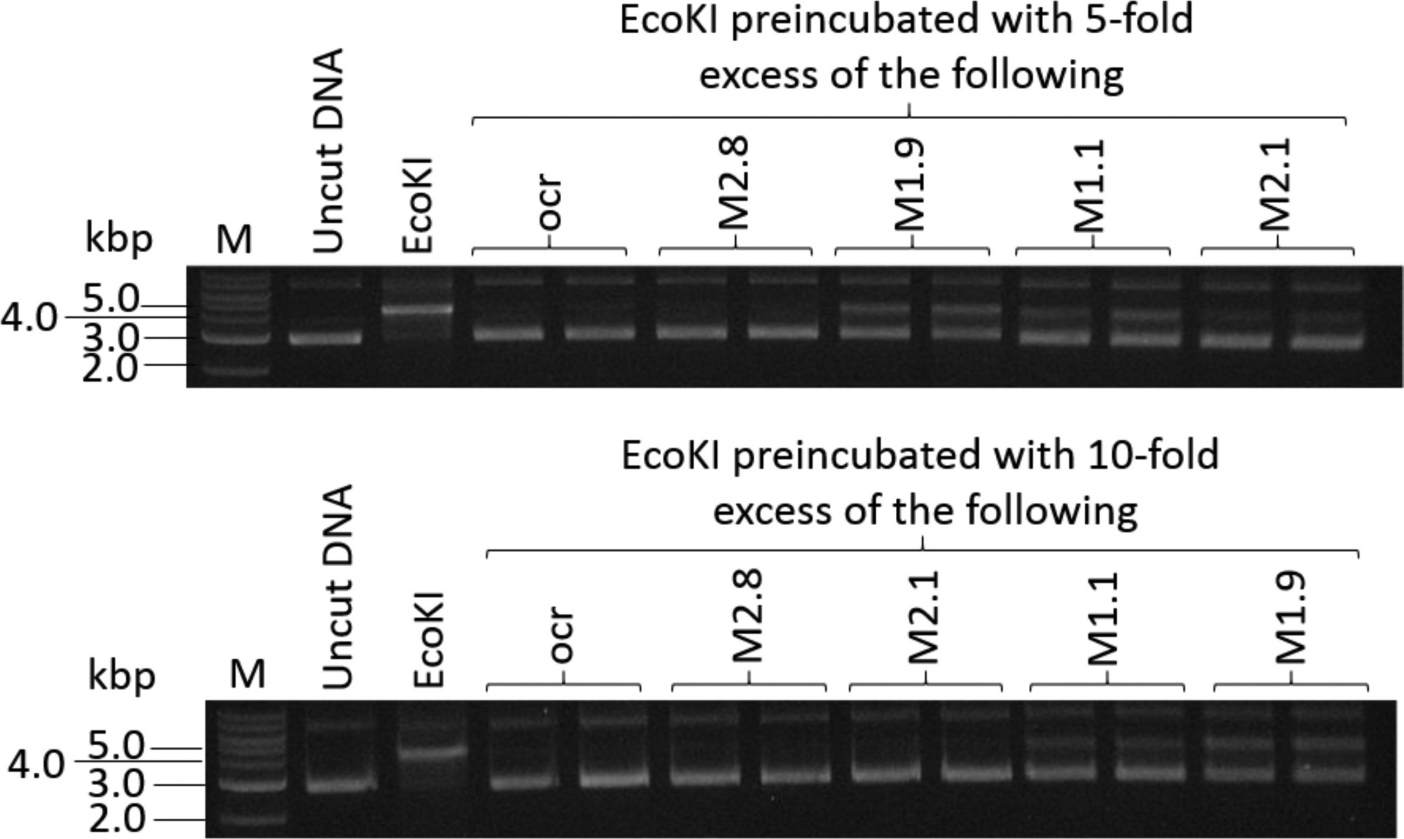
Endonuclease assay to analyse the inhibition of EcoKI nuclease activity by the pocr mutants. Unmethylated pBRsk1 was digested with EcoKI to give a linear species of ∼4.4 kb. The experiment was also repeated using EcoKI that had been preincubated with either Ocr or a pocr mutant prior to the reaction. In each case the reactions were performed in duplicate. The ratio of EcoKI to Ocr or pocr was either 1:5 (top gel) or 1:10 (bottom gel). The reaction products were resolved on a 0.9% agarose gel. Size marker; 1 kbp ladder (NEB).

*In vitro* nuclease assays were performed to analyse the level of inhibition exerted by the mutated forms of pocr (Figure 2). Unmethylated pBRsk1, containing a single EcoKI recognition sequence, was used as substrate. A 10-fold excess of Ocr/pocr was pre-incubated with EcoKI prior to the addition of plasmid substrate and cofactors. Linearisation of the plasmid DNA was assessed by subjecting the reaction mixture to agarose gel electrophoresis. Using a molar ratio of 1:10 of nuclease to antirestriction protein, complete inhibition of EcoKI by Ocr, M2.8 and M2.1 was observed. By contrast, the pocr mutants M1.1 and M1.9 displayed only partial inhibition of the endonuclease. The experiment was then repeated using a reduced molar ratio of 1:5 nuclease to antirestriction protein. Under these conditions, partial inhibitory activity was observed for pocr mutant M2.1 as well as M1.9 and M1.1. However, the mutant M2.8 together with wild-type Ocr retained full inhibitory activity.

### Analysis of further mutants from the ISOR libraries

The recovery of only four active protein sequences from the millions of sequences generated by the ISOR procedure was disappointing and suggests that the evolutionary path from pocr to Ocr is difficult. Thus we decided to return to the first three libraries generated and to select at random a group of 65 sequences for further analysis using our range of *in vivo* techniques (Supplementary Table S3). Eleven of the group proved to give irreproducible results when growing on media containing 20 μg/ml 2AP, sometimes growing and sometimes not, so these were not analysed further. This group contained two, seven and two members from libraries 1, 2 and 3 respectively.

**Figure 3.**
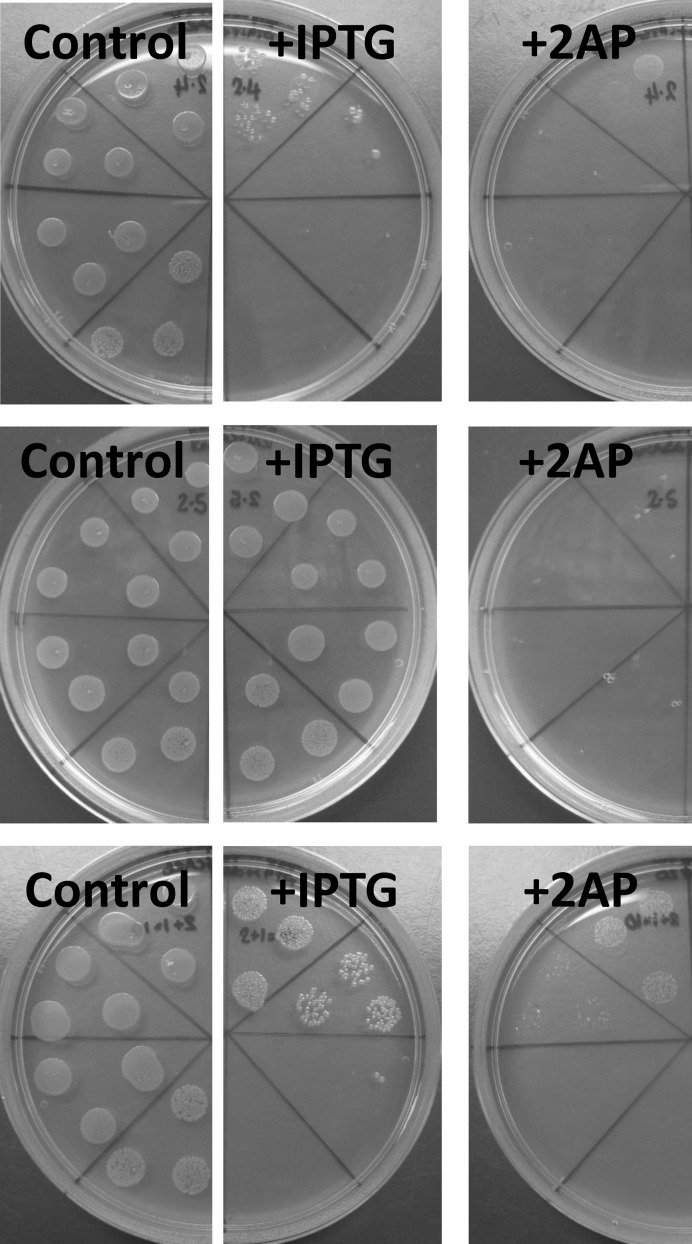
Cell growth of *E. coli* NM1041 (r^+^) after transformation with different mutants from the ISOR library. Top: a partially active mutant (mutant 2.4). Middle: an inactive mutant (mutant 2.5). Bottom: a toxic mutant (mutant 21+10). From left to right each panel shows growth on LB-agar supplemented with ampicillin (50 μg/ml), with 50 μg/ml IPTG and ampicillin, and lastly with 50 μg/ml IPTG, 20 μg/ml 2AP and ampicillin. Serial dilutions of cultures were spotted in triplicate in an anticlockwise or clockwise fashion.

The remaining 54, made up of 21, 15 and 18 representatives from libraries 1, 2 and 3 respectively, gave reproducible results in the 2AP assay showing several different behaviors in a strain expressing HsdR, namely, limited growth in the presence of IPTG and even more limited growth in the presence of 20 μg/ml 2AP (without filamentation—see later), growth in the presence of IPTG but not when 20 μg/ml 2AP was also present and limited growth in the presence of IPTG and even more limited growth in the presence of 20 μg/ml 2AP (with filamentation – see later) (Figure 3). We term these three situations as Partially Active (PA), Inactive (IN) and Toxic, respectively. Previously we have found fully active mutants and three partially active mutants, Mut12, Mut16 and Ocr/pocr, but the three partially active mutants were not able to grow at all in the presence of 20 μg/ml 2AP (15). These partially active mutants are therefore distinct from the current PA mutants, which do grow a little in the presence of 2AP and are therefore listed as PA* in Supplementary Table S3.

The ISOR mutants in groups PA, IN and Toxic were tested using the antirestriction and antimodification assays and this revealed that, apart from one instance, these three groups possessed no antirestriction or antimodification activity (Supplementary Table S3). The mutant 3.11 showed full antirestriction and antimodification activity (antirestriction value of 0.37 and antimodification value of 1.9 × 10^−4^ for comparison to values in Table 1) despite being classified as Toxic in the presence of IPTG. Leaky expression in the absence of IPTG has been found previously to be sufficient for antirestriction and antimodification activity (11) so this apparently contradictory behaviour of mutant 3.11 can be reconciled. The antirestriction and antimodification activity of this mutant indicates that the protein is folded.

**Figure 4.**
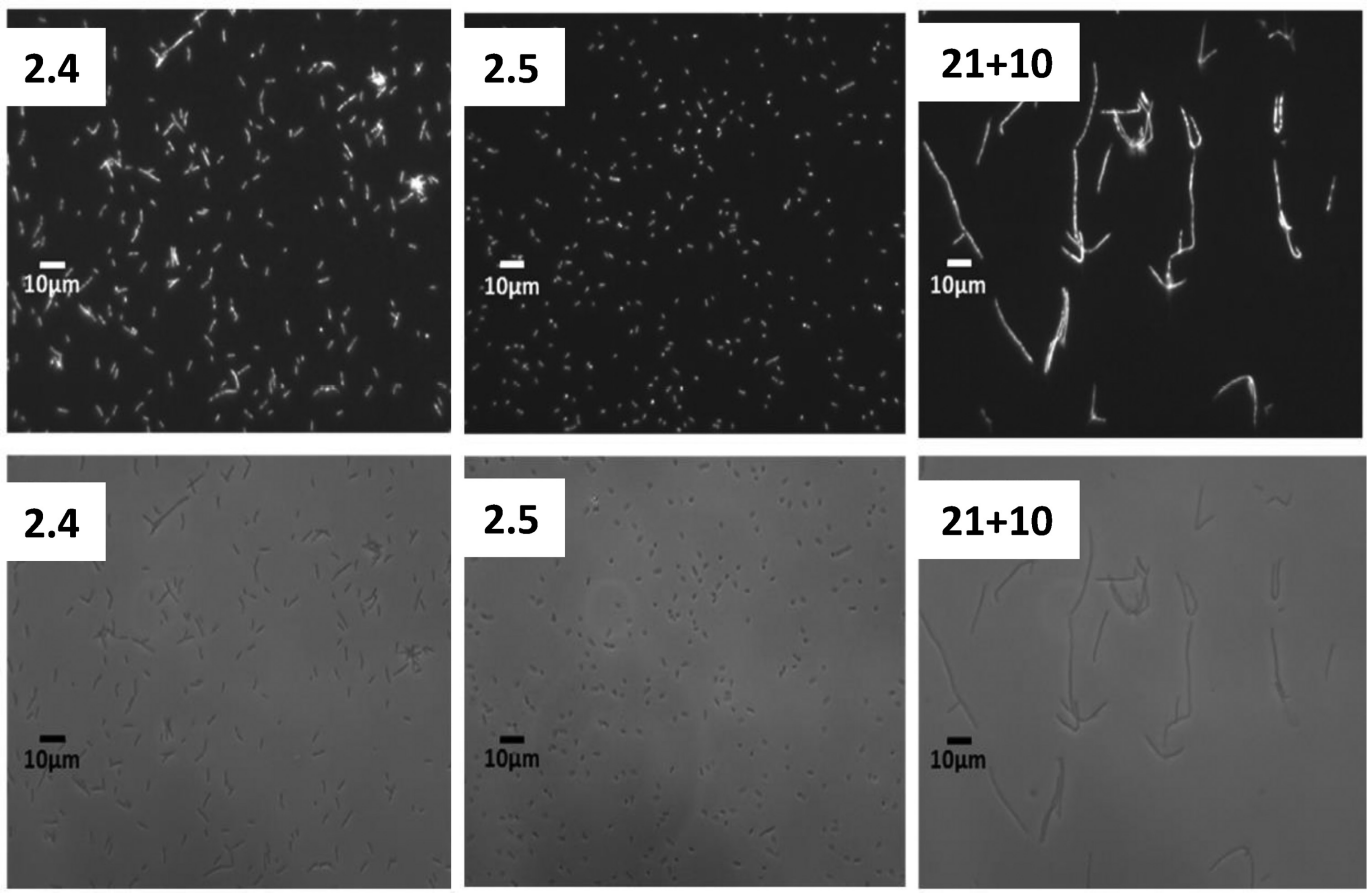
Microscopy images of *E. coli* NM1041 (r^+^) transformed with different mutants from the ISOR library. The top row shows DAPI fluorescence from stained chromosomes while the bottom row shows phase contrast images of the whole cells. From the left to right the cells are transformed with a partially active mutant (mutant 2.4), an inactive mutant (mutant 2.5) and a toxic mutant (mutant 21+10).

Microscopy revealed that the IN and Toxic mutants induced filamentation indicative of a deleterious effect on cell division and suggestive that they are interacting with other proteins (Figure 4 and Supplementary Table S3). This occurred in both *hsdR*^−^ and *hsdR*^+^ strains.

**Figure 5.**
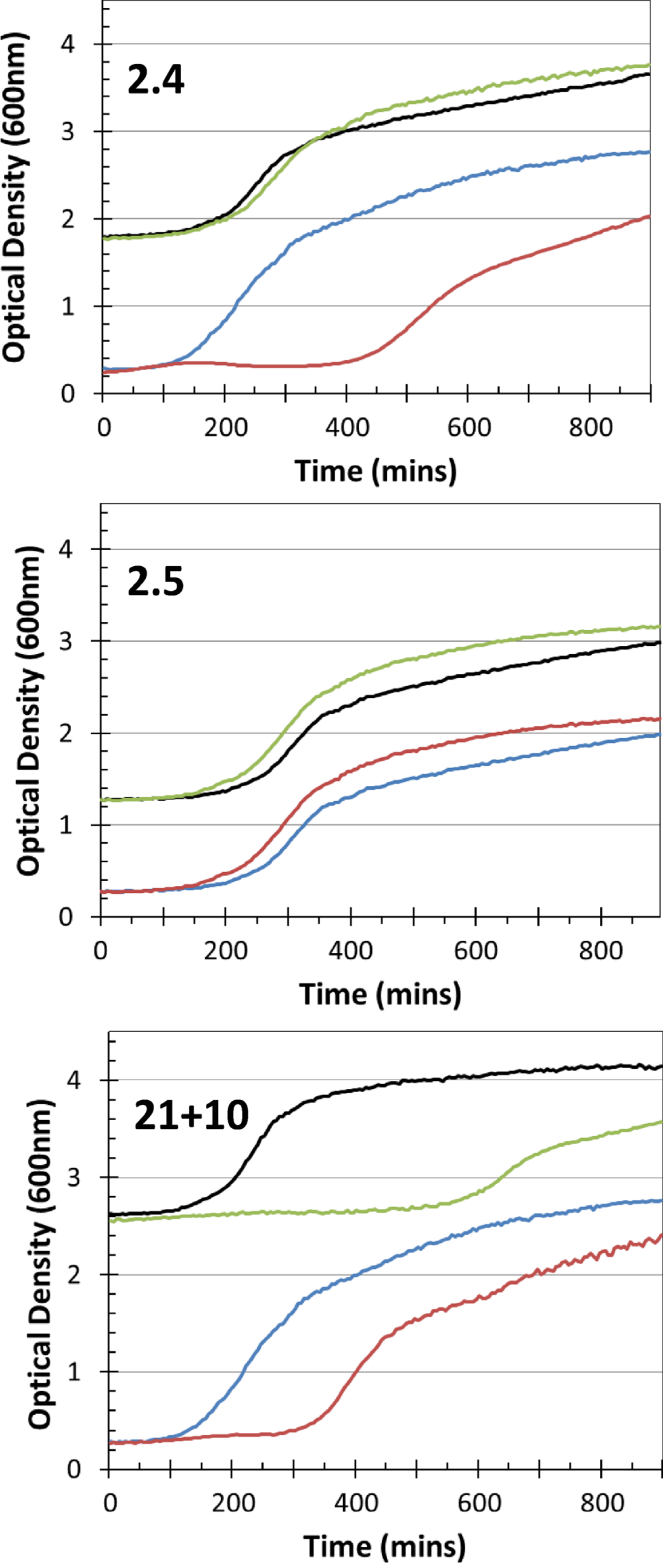
Growth curves of *E. coli* transformed with different mutants from the ISOR library. Top: a partially active mutant (mutant 2.4). Middle: an inactive mutant (mutant 2.5). Bottom: a toxic mutant (mutant 21+10). *E. coli* NM1261 (r-) in green is supplemented with 50 μg/ml IPTG and in black is without IPTG. *E. coli* NM1041 (r^+^) in red is supplemented with IPTG and in blue is without IPTG. The *E. coli* NM1261 curves are offset from the zero.

Analysis of growth curves for the three main groups of mutants in *hsdR*^−^ and *hsdR*^+^ strains showed that the PA mutants grew normally in the absence of HsdR but grew slowly in its presence, thus indicating that the mutants are capable of interacting with HsdR even though not sufficiently strongly to inhibit restriction in the antirestriction assay (Figure 5, Supplementary Table S3). These findings suggest this group of mutant proteins are folded *in vivo*. The IN group of mutants had no effect on the growth curves irrespective of whether HsdR was present or not (Figure 5, Supplementary Table S3). Thus, it is not clear from this experiment whether these mutant proteins are capable of folding. However, the observation of filamentation (Figure 4) suggests that they are capable of interacting with other proteins in the cell so they may be folded. In addition, we have previously found that inactive mutant forms of Ocr can be purified and are folded (15). Thus, we suggest that at least some members of the IN group are folded. The mutants in the Toxic group showing no antiRM activity demonstrated slowed cell growth in both *hsdR*^−^ and *hsdR*^+^ strains (Figure 5, Supplementary Table S3). This observation indicates that these mutant proteins are folded and capable of interacting with HsdR as well as other protein species within the cell.

Taken together, we conclude that all of the PA and Toxic mutants selected for analysis are folded *in vivo*. Moreover, some of these mutant forms of pocr are capable of interacting with the EcoKI Type I enzyme and others can also interact with alternative proteins to interfere with cell division. We also conclude that a non-zero proportion of the IN mutants are folded.

### Amino sequence analyses

Sequence alignments were performed on each group of ocr/pocr mutant forms, namely Active, PA*, PA, Inactive and Toxic. The complete alignments for each group of variants are given in Supplementary Figure S7. Although it is apparent that sequences associated with non-active variants of the wild-type Ocr protein tend to have fewer acidic residues, no obvious straightforward pattern associated with the groupings derived by our experimental results is immediately discernible. A further alignment derived from a BLASTP search of non-redundant protein sequences using the wild type Ocr sequence was performed, Supplementary Figure S8. This alignment revealed that homologues to Ocr contained very similar patterns and numbers of acidic residues to Ocr.

**Figure 6.**
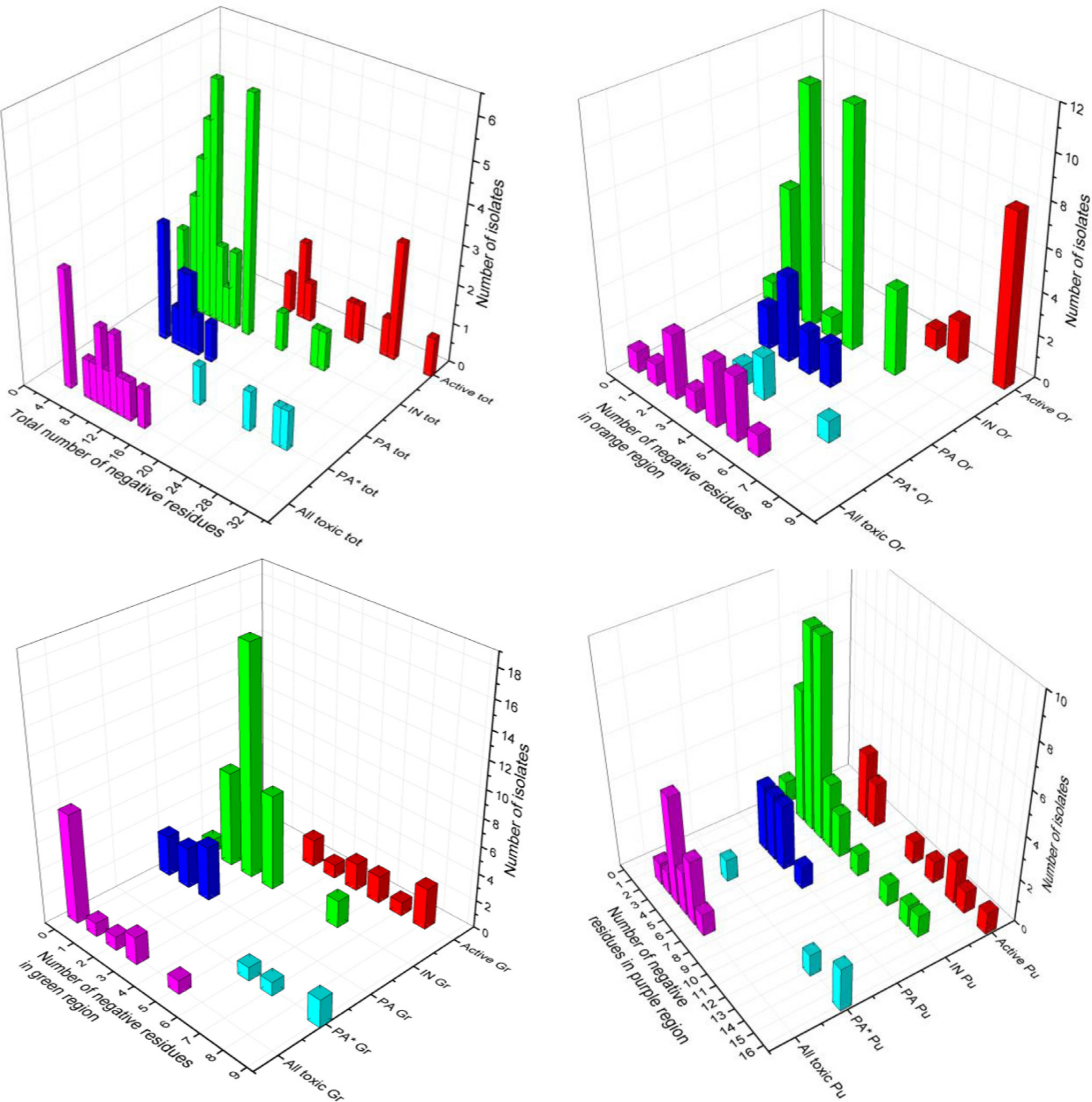
Bar charts showing the number of sequences analysed in each group of activities having a particular number of negative charges in various structural regions of Ocr. The bars are coloured as follows: Active (red), Inactive (IN, green), partially active (PA, blue), partially active from reference 15 (PA*, cyan) and toxic (magenta).

**Figure 7.**
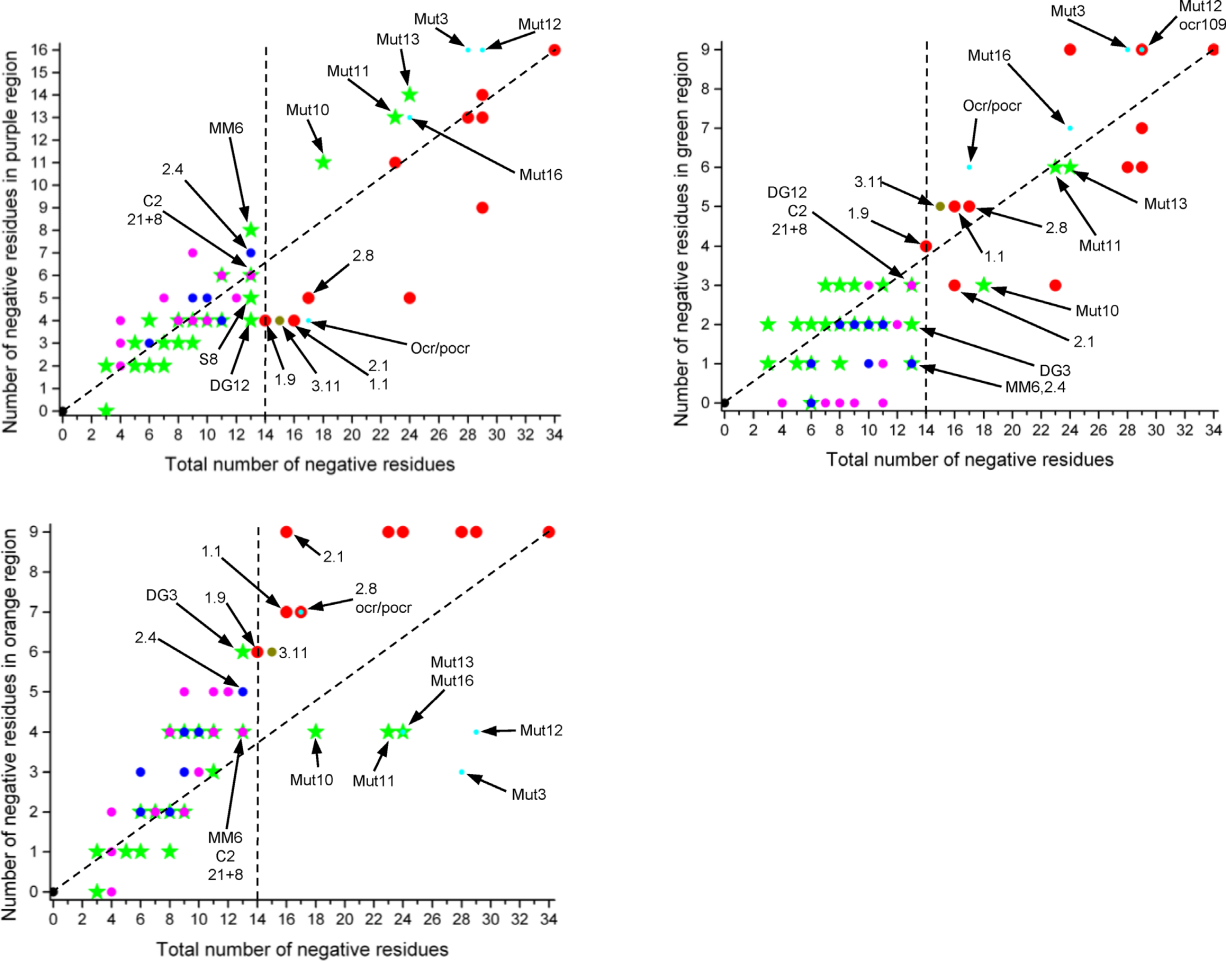
Plots of the number of negative charges in the three regions defined in Figure 1b against the total number of negative charges in the whole sequence for each Ocr and pocr variant. The points are coloured as follows: Active (red), Inactive (green), Partially Active (blue), Partially Active from reference 15 (PA*, cyan), Toxic (magenta), Toxic mutant 3.11 (olive) and pocr (black).Various mutants are highlighted including the MutX mutants from reference 15. The vertical dashed line indicates a total negative charge of −14 while the other dashed line joins pocr to wild type Ocr.

To pull out any trend in the arrangement or number of negative residues in a given Ocr or pocr variant, a number of further analyses were performed. Firstly, the number of negative residues in the complete sequence, in the three sections mimicking the different parts of the DNA target shown in Figure 1b, and in the two loops (loops 1 and 2) were determined for each Ocr and pocr variant, Supplementary Table S3. Secondly, the number of sequences in each group containing a given number of negative residues in a particular region was plotted on a bar chart, Figure 6. Lastly, the number of negative charges for each Ocr or pocr variant in each region was plotted against the total number of negative charges in the Ocr or pocr variant, Figure 7.

The bar charts, Figure 6, split the various groups of activities with it being particularly clear that an active ocr variant requires at least 14 negative residues in total and that these should be located in the ‘orange’ region shown in Figure 1b. The other groups of activities tend to overlap with each other so no further trend is very obvious in these bar charts. Figure 7 pulls out some further features of the number of negative residues to be found in each group of activities. To be active, in addition to at least 14 negative residues in total, Ocr requires an excess of charges in the orange region (at least 6 of a possible 9) and no more than average in the purple region (no >47% of the total). The number of negative charges in the green region is not too important as long as it is at least three. The active variants found in the ISOR library are all close to the boundary between inactivity and activity while those engineered previously (15) are closer to the wild type Ocr charge composition as expected from their biased construction method. This biased construction method also places some inactive variants (Mut10, Mut11 and Mut13) and the PA* variants (Mut3, Mut12, Mut16 and ocr/pocr) within the space occupied by the active Ocr variants.

The unusual pocr variant, 3.11, which shows antiRM activity but also toxicity lies within the group of active variants isolated from the ISOR library. A range of Inactive, PA and Toxic variants from the ISOR library possess 13 negative charges and lie just below the boundary for Ocr activity (MM6, C2, 21+8, 2.4, DG3 and DG12). These particular mutant forms of Ocr show fewer than expected charges in the green region, more than expected in the orange region and no correlation in the purple region.

## DISCUSSION

The DNA mimicry of Ocr, which is crucial for its ability to act as an antirestriction protein, comprises two main features; namely, shape and charge. Here, we have investigated the charge mimicry of Ocr. The Ocr protein is replete with acidic residues, which generate a highly negatively charged surface that mimics the phosphate backbone of the DNA duplex. ITC data for the interaction between Ocr and M.EcoKI under different salt conditions is consistent with the idea that the binding is driven, at least to a large extent, by electrostatics (13). Previously, we have investigated the charge mimicry by removing charge from the wild type Ocr protein focussing either on one or two residues at a time (14) or on contiguous blocks of charge (15) and our results indicated that loops 1 and 2 included within the regions mimicking the DNA target of the RM enzyme were important for activity (Figure 1). However, this methodology is strongly biased by our knowledge of the protein structure. Here, we have approached the evolution of Ocr from the opposite direction by starting from a gene encoding no acidic residues, but with the remainder matching Ocr, and then subjecting the gene to directed evolution until activity was detected. In this way the two approaches will overlap to reveal at what point activity appears.

In order to gradually evolve the pocr gene in the desired fashion, we chose to utilise a modified version of the ISOR methodology (35,36). We were able to gradually mutate the pocr gene and select for antirestriction activity after each round of ISOR. In all, three rounds of mutagenesis were required before clones showing antirestriction activity could be detected. Seven individual clones (M1.1, M1.4, M1.7, M1.9, M2.1, M2.8 and M2.11) were selected but only four, M1.1, M1.9, M2.1 and M2.8 were unique. These protein sequences were folded and active in a range of *in vivo* and *in vitro* experiments. Thus, the evolutionary process to introduce negative charges into pocr had generated mutants of Ocr that overlap with those made by removing negative charges from the wild type Ocr. The effectiveness of the pocr proteins as antirestriction proteins follows the order: wild type Ocr = M2.8 > M2.1 > M1.1 > M1.9.

**Figure 8.**
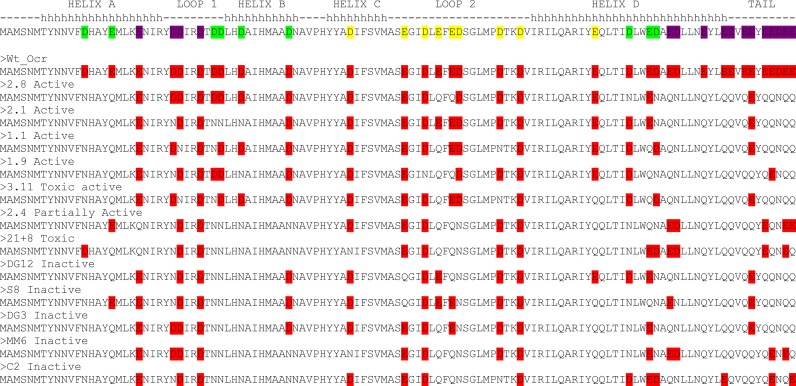
Sequence alignment of the active pocr clones selected from the third round of directed evolution and those found to be inactive, toxic or partially active but being very close in charge distribution to active mutants. Acidic residues are highlighted by red shading. The secondary structure elements of Ocr (helix = h, loop = dash) and the regions corresponding to different parts of the DNA target (highlighted in green, orange and purple) as shown in Figure 1b are also given.

A sequence comparison of these pocr clones along with that of Ocr is shown in Figure 8. The four pocr mutant sequences display some striking similarities to one another. Firstly, the number of acidic residues in each pocr mutant is similar i.e., 14, 16, 16 and 17 for M1.9, M1.1, M2.1 and M2.8, respectively. At least 50% of the acidic residues introduced into the four pocr clones are clustered in the two loop regions of Ocr (Figures 1a and Figure 8). Sequence alignments reveal M2.1 contains all seven acidic residues within loop 2, and mutant M2.8 includes all five acidic residues in loop 1 (Figure 8). These findings support the proposal that acidic residues in the two loop regions (particularly loop 2) are important for antirestriction activity as previously suggested on the basis of site directed mutagenesis studies (15). If from these results and the structural features highlighted in Figure 1, we propose that the acidic residues in the two loops or alternatively, the green and orange regions identified as mimicking the sequence-specific and the central non-specific parts of the Type I RM enzyme target sequence, are particularly significant for the interaction of Ocr with Type I RM enzymes, then one might anticipate that these amino acids would be well conserved. A sequence alignment of Ocr homologues reveals that there is indeed a very high degree of conservation of acidic residues in these loops and DNA-mimicking regions (Supplementary Figure S8).

However, it must be borne in mind that pocr itself does not appear to fold into a stable protein. Hence, some of the amino acid substitutions introduced into selected pocr mutants during the process of directed evolution will be structurally important. Initially, we focussed on evolving antirestriction activity, but only a small number of positive mutants were isolated. To fill in the gap from inactive pocr to active mutants M1.1, M1.9, M2.1 and M2.8, we re-examined the three libraries produced in the first rounds of ISOR. A survey of a range of pocr mutants from these libraries revealed several behaviours falling short of full antirestriction activity, which were grouped as Inactive, Toxic or partially active (PA) (Figures 3-5, Supplementary Table S3). Given that most of these mutant forms of pocr elicited a significant effect on the growth characteristics of their hosts, it is clear the proteins are being expressed and are able to fold. The analysis of fully active pocr and Ocr mutants and homologues proteins indicates that the amount of charge in the regions mimicking the DNA target sequence and the non-specific central part of the target (the green and orange regions of Figure 1b, respectively) and the overall total charge are important for activity, Figures 6 and 7.

Figure 7 shows plots of all of our mutants derived from Ocr (15) and from pocr using axes for total negative charge and total negative charge in various defined regions of the structure. These plots reveal a continuum of mutants stretching from the inactive pocr to the fully active Ocr. The three groups of mutants, inactive, PA and toxic, lacking antirestriction and antimodification activity all overlap with each other indicating that the fine details of the location of the negative charges can have a large effect on the behaviour of the polypeptide. These three groups are separate from the active mutants.

Of particular interest is the region at which the four active pocr mutants found in this work appear. In the middle of this group lies a very unusual pocr mutant, 3.11, which showed full antirestriction and antimodification activity but whose overexpression resulted in toxicity and cell death. Just next to this active group and mutant 3.11 lie several Inactive, PA and Toxic variants. A sequence alignment of these variants with 3.11 and the active Ocr sequences is given in Figure 8. Crucially all these inactive, PA or toxic mutants are lacking negative charges on Helix B in the green region of the structure and lack aspartate at position 88 (except DG12). This E88 location is conserved in active forms of the mutants and in naturally occurring relatives of Ocr (Figure 8, Supplementary Figure S8 and Supplementary Table S3). Thus, based on sequence analysis; (i) activity requires sufficient negative charge in the green and orange regions, and (ii) toxicity is avoided by maintaining aspartate at position 88. The avoidance of toxicity would also provide the evolutionary pressure to move away from this small region of the plots in Figure 7 by acquiring further negative charge.

**Figure 9.**
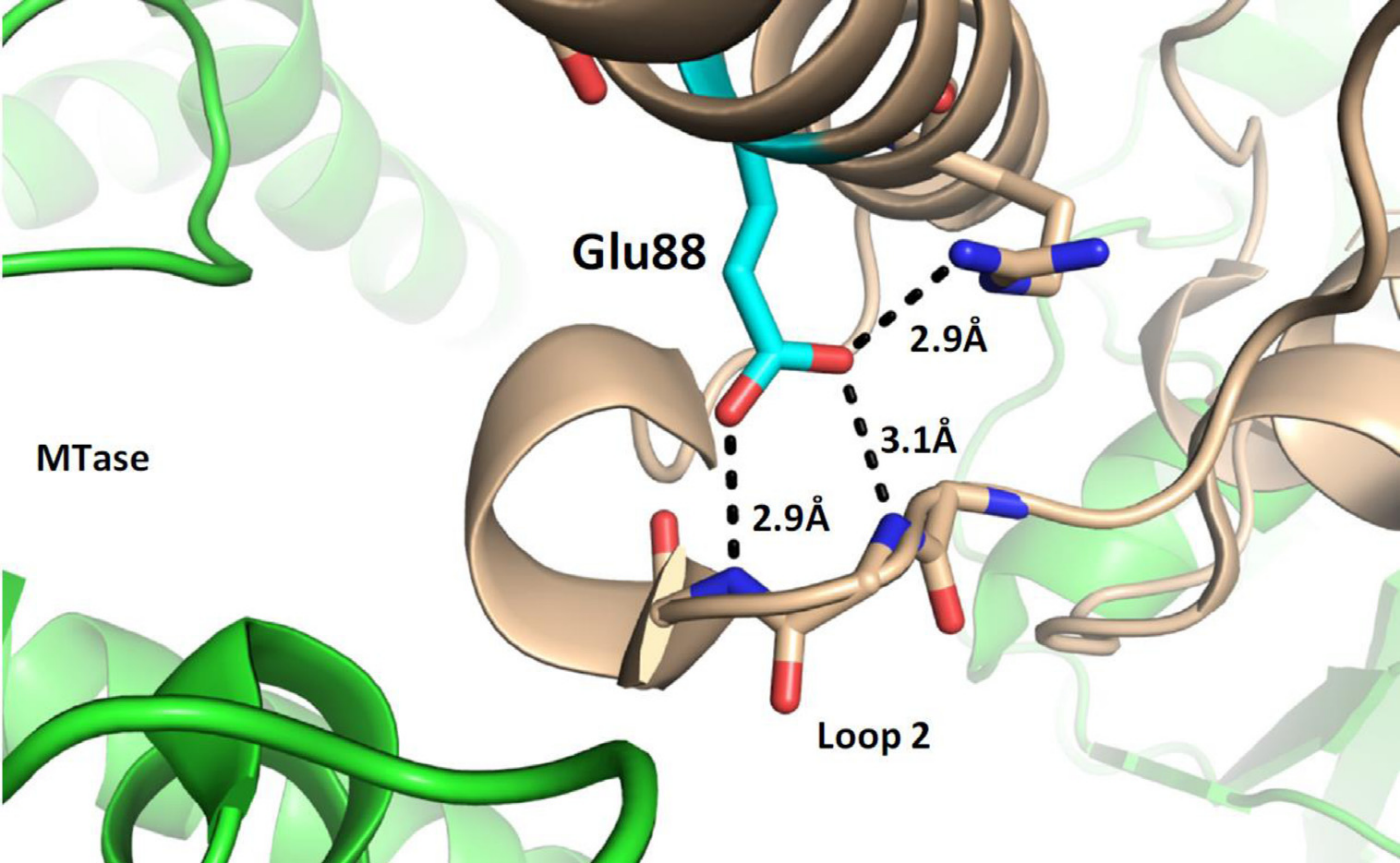
The structural model of the complex of M.EcoKI (green) bound to Ocr (light brown, 26) focussing on the possible hydrogen bonding interactions in the Ocr structure between E88 (cyan) in Helix D with the amide groups of the main peptide chain of Loop 2 (PDB:2Y7C). The residues and main chains are displayed using sticks and the hydrogen bonds using black dotted lines. The bond lengths are all ∼3 Å.

Examination of the crystallographic structure of Ocr shows that E88, which is part of Helix D, can potentially make several hydrogen bonds to residues in loop 2 (Figure 9). If the ability to make these hydrogen bonds is removed, as in the toxic mutant 3.11, then loop 2 will be much more flexible. This may allow loop 2 to form unwanted interactions with proteins involved in cell division and hence cause growth problems for the host cell. It is worth recalling that Ocr has been shown to bind to the RNA polymerase in *E. coli* so the ability to form extraneous interactions is not unprecedented (40).

The number of potential variants of ocr containing 34 possible negative or neutral amino acids is 2^34^ or 1.7 × 10^10^ and we screened ∼1.2 × 10^6^ of this number. In our screen we found only four unique active sequences or 0.0003% of the 1.2 × 10^6^ sequences screened. If this 0.0003% of active sequences is true for the 2^34^ variants then we expect the 2^34^ variants to include 0.0003% of 2^34^ active sequences or ∼2^16^ active sequences indicating that an active Ocr sequence should contain about 16 negative charges. This is approximately the same as found experimentally. Obviously Ocr has not really evolved from pocr but will have started from some other location in the plots shown in Figure 7 and most probably with an ‘average’ number of negative and positive side chains (12 of each would be expected rather than the 34 and 6 respectively actually observed per monomer). However, whatever the evolutionary origins of Ocr, the surfeit of acidic residues ensures that both modification and restriction are efficiently inhibited and toxicity is avoided.

In summary, a detailed examination of our data shows that to obtain an active antirestriction and antimodification Ocr protein capable of being expressed without deleterious effects on the host cell one has to achieve several conditions as follows:

The pI must be 4.5 or less.
The number of negative charges in the orange region must be at least 6.
In the purple region, there should be no more than 47% of the total number of negative charges.
The green region should contain at least three negative charges.
The total number of negative charges on the whole sequence must be at least 14.
Position 88 must be aspartate to form hydrogen bonds to loop 2 thereby constraining its conformation and removing its ability to interact with other proteins in the cell involved in cell division.

## Supplementary Material

SUPPLEMENTARY DATA
