# Supplementary material for: The evolutionary pathway from a biologically inactive polypeptide sequence to a folded, active structural mimic of DNA
Source: Nucleic Acids Res. 2016 Apr 19;44(9):4289–303. doi: 10.1093/nar/gkw234 (PMC4872106; doi:10.1093/nar/gkw234)
Supplement: SUPPLEMENTARY DATA [file supp_44_9_4289__index.html]

The evolutionary pathway from a biologically inactive polypeptide sequence to a folded, active structural mimic of DNA — SUPPLEMENTARY DATA 

# The evolutionary pathway from a biologically inactive polypeptide sequence to a folded, active structural mimic of DNA

## SUPPLEMENTARY DATA

- SUPPLEMENTARY DATA
- SUPPLEMENTARY DATA
