# Supplementary material for: The evolutionary pathway from a biologically inactive polypeptide sequence to a folded, active structural mimic of DNA
Source: Nucleic Acids Res. 2016 Apr 19;44(9):4289–303. doi: 10.1093/nar/gkw234 (PMC4872106; doi:10.1093/nar/gkw234)
Supplement: SUPPLEMENTARY DATA [file supp_gkw234_nar-00696-h-2016-File005.docx]

**The evolutionary pathway from a biologically inactive polypeptide sequence to a folded, active structural mimic of DNA.**

Nisha Kanwar, Gareth A. Roberts, Laurie P. Cooper, Augoustinos S. Stephanou & David T.F. Dryden*.

EaStCHEM School of Chemistry, University of Edinburgh, The King’s Buildings, Edinburgh EH9 3FJ, UK

* Author for correspondence:

david.dryden@ed.ac.uk, Tel. +44 131 650 4735

**Supplementary Figures**

Figure S1

Nucleotide sequence of the pocr gene (synthesised by GeneArt). The corresponding protein sequence is also shown. The start codon is highlighted in grey shading. The NdeI restriction recognition sequence, which facilitated convenient cloning, is underlined. Asn and Gln residues in pocr that were targeted for mutation to Asp and Glu, respectively, are highlighted in yellow.

H M A M S N M T Y N N V F N H A Y Q M L K Q N I R Y N

1 CATATGGCTATGTCTAACATGACGTACAATAACGTTTTTAATCACGCATATCAGATGCTGAAACAGAACATTCGCTACAA 80

1 GTATACCGATACAGATTGTACTGCATGTTATTGCAAAAATTAGTGCGTATAGTCTACGACTTTGTCTTGTAAGCGATGTT 80

N I R N T N N L H N A I H M A A N N A V P H Y Y A N

81 TAACATTCGTAATACCAACAACCTGCACAACGCCATCCACATGGCCGCGAACAACGCAGTCCCACACTACTACGCGAACA 160

81 ATTGTAAGCATTATGGTTGTTGGACGTGTTGCGGTAGGTGTACCGGCGCTTGTTGCGTCAGGGTGTGATGATGCGCTTGT 160

I F S V M A S Q G I N L Q F Q N S G L M P N T K N V I

161 TCTTCAGCGTTATGGCTTCTCAGGGTATCAACCTGCAGTTCCAGAACTCCGGCCTGATGCCGAACACCAAAAACGTAATC 240

161 AGAAGTCGCAATACCGAAGAGTCCCATAGTTGGACGTCAAGGTCTTGAGGCCGGACTACGGCTTGTGGTTTTTGCATTAG 240

R I L Q A R I Y Q Q L T I N L W Q N A Q N L L N Q Y L

241 CGTATTCTGCAGGCTCGTATCTACCAGCAGCTGACTATCAACCTGTGGCAAAACGCGCAGAACCTGCTGAACCAGTATCT 320

241 GCATAAGACGTCCGAGCATAGATGGTCGTCGACTGATAGTTGGACACCGTTTTGCGCGTCTTGGACGACTTGGTCATAGA 320

Q Q V Q Q Y Q Q N Q Q *

321 GCAACAGGTGCAGCAATATCAGCAGAACCAACAGTAA 357

321 CGTTGTCCACGTCGTTATAGTCGTCTTGGTTGTCATT 357

Figure S2

Sequence analysis of the initial four sublibraries (Lib 1-4). A total of 10 individual clones from each of the four sublibraries were picked and the pocr insert DNA was sequenced. Either eight or nine pocr codons were targeted in each of the four libraries (see Table S1). The percentage coverage of each codon is displayed.


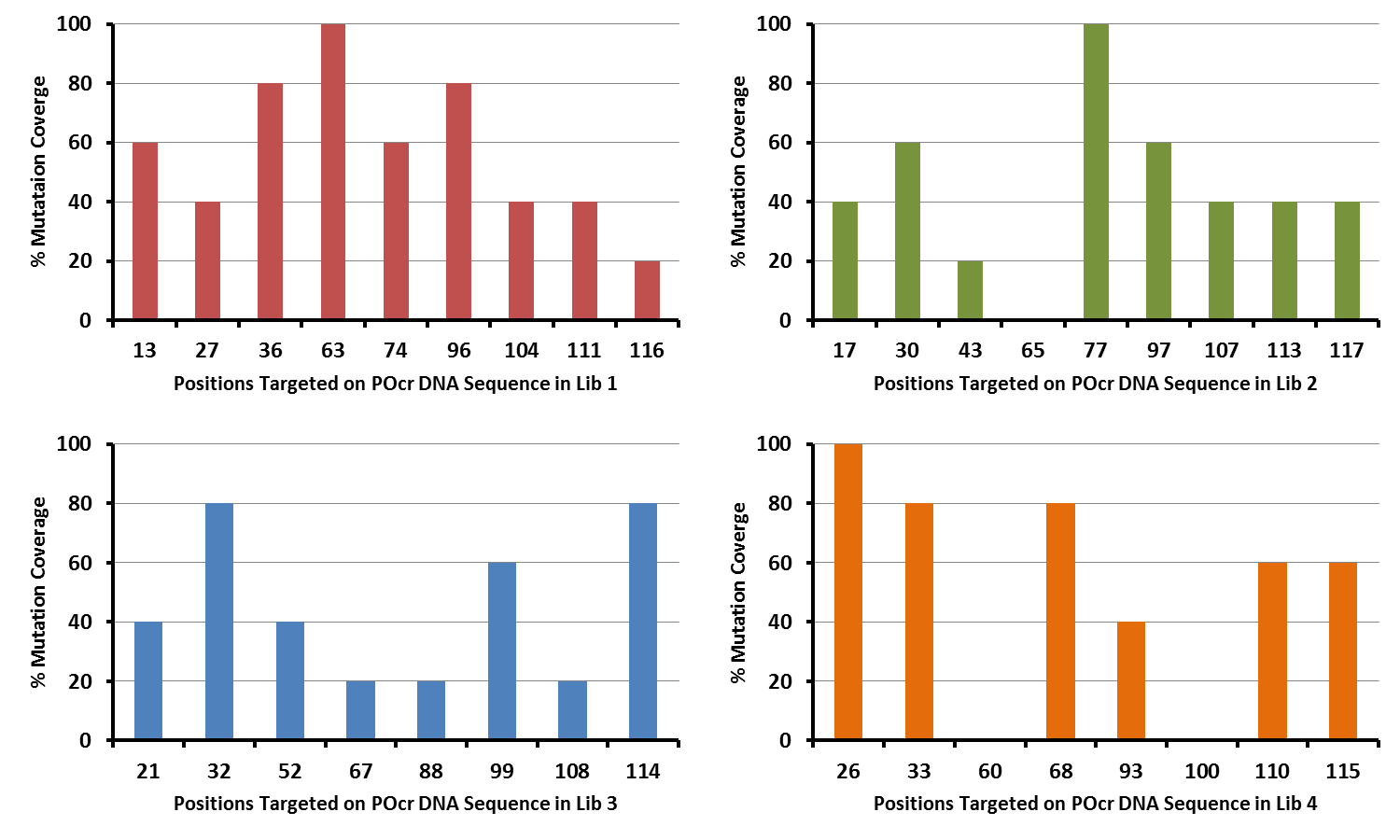


Figure S3

Far UV-CD analysis of Ocr along with the selected pocr mutants. In each case, the protein concentration was adjusted to 30 µM. The data indicate that the secondary structure of pocr mutant proteins is not significantly different from that of Ocr.


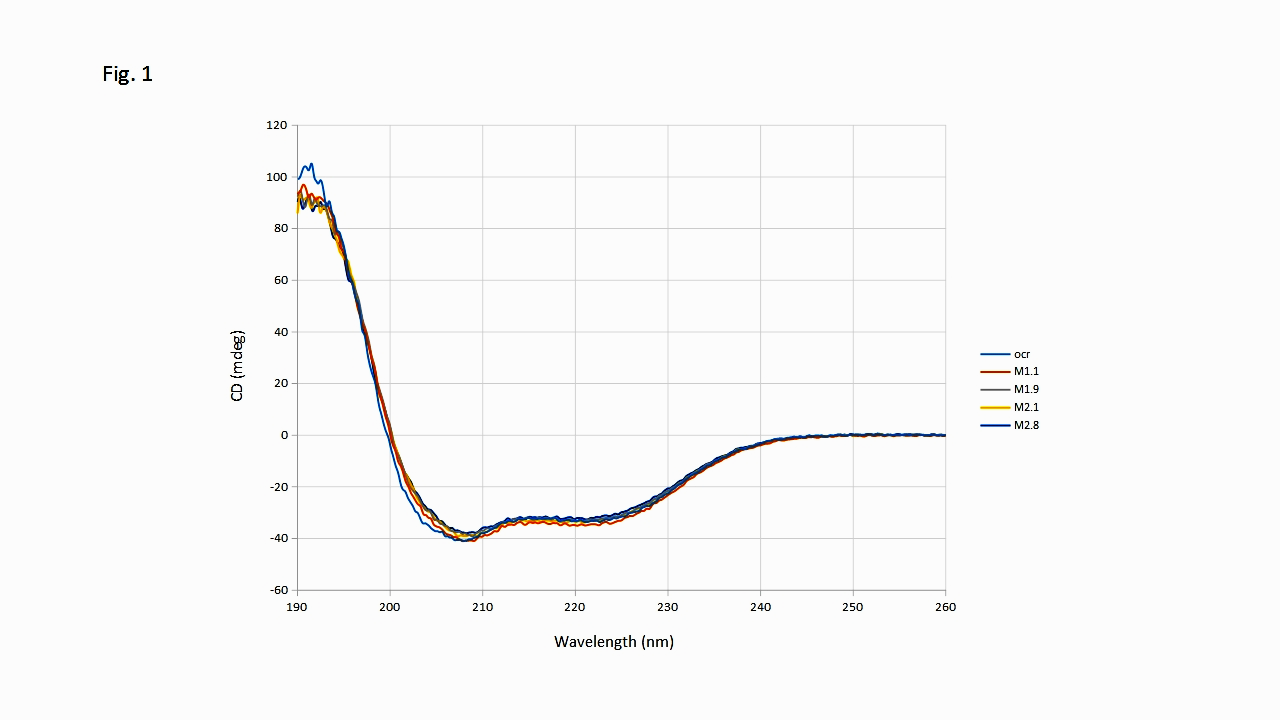


Figure S4

Glutaraldehyde crosslinking of the purified pocr mutants. Purified Ocr was used as a positive control. Dilute samples were crosslinked with glutaraldehyde and the protein was then precipitated with trichloroacetic acid and analysed by SDS-PAGE (4-12% acrylamide gel). In each case, a proportion of the protein migrates as a dimer species (26 kDa) after crosslinking. Any crosslinked species must form by dimerisation across the dimer interface, which makes up a relatively small proportion of the protein (based on the 3D structure of Ocr, PDB 1S7Z). Hence, the majority of the sample runs as a monomer (13 kDa) even after crosslinking. The crosslinked Ocr species migrates through the gel at a different rate from that of the pocr mutants. This anomalous migration may be related to the highly acidic nature of Ocr by comparison to the pocr mutants. A sample on uncrosslinked Ocr was run on the gel for comparison. The two images are of the same gel but the contrast is increased in the right hand image to make the dimer bands more prominent.


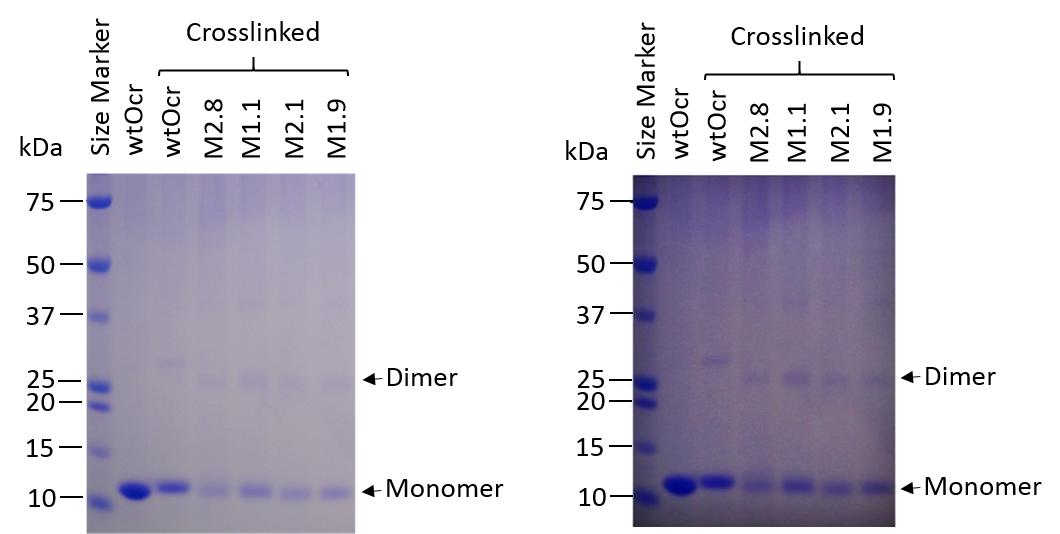


Figure S5

Thermal stability of Ocr and the selected pocr mutants (M1.1, M1.9, M2.1 or M2.8) was examined using a quantitative real-time thermocycler. The protein samples were mixed with SYPRO orange dye and the fluorescence emission was measured as a function of temperature. The peaks correspond to the thermal denaturation of the protein. The thermal denaturation for Ocr, M2.1, M2.8 and M1.1 are similar (54-57°C) whereas the peak for M1.9 shifted to a higher temperature (67°C).


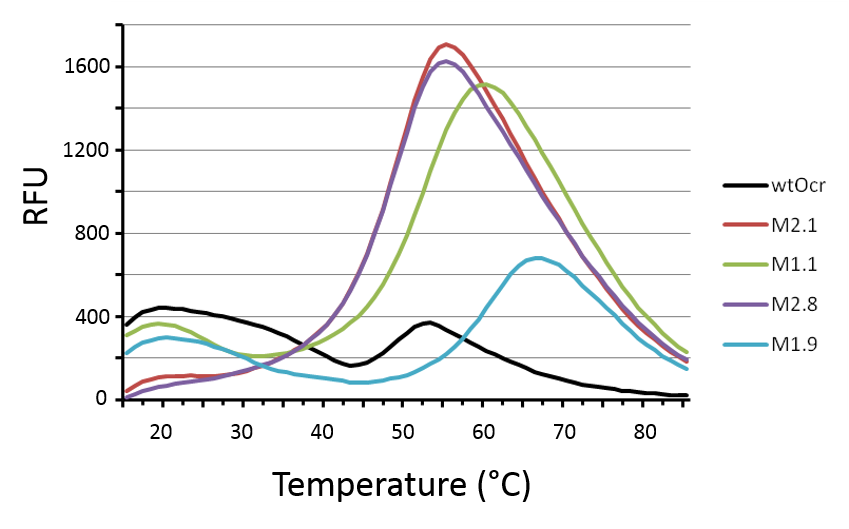


Figure S6

Isothermal calorimetry analysis for the interaction between M.EcoKI and selected pocr mutants or Ocr for comparison. In each case, Ocr or pocr mutant (30 µM dimer) was injected into the cell containing M.EcoKI (3 µM). Calorimetric data were converted into differential binding curves by integration of the resultant peaks. Data were then fitted to a single-site binding model using the Microcal LLC Origin software to determine the parameters in the table.


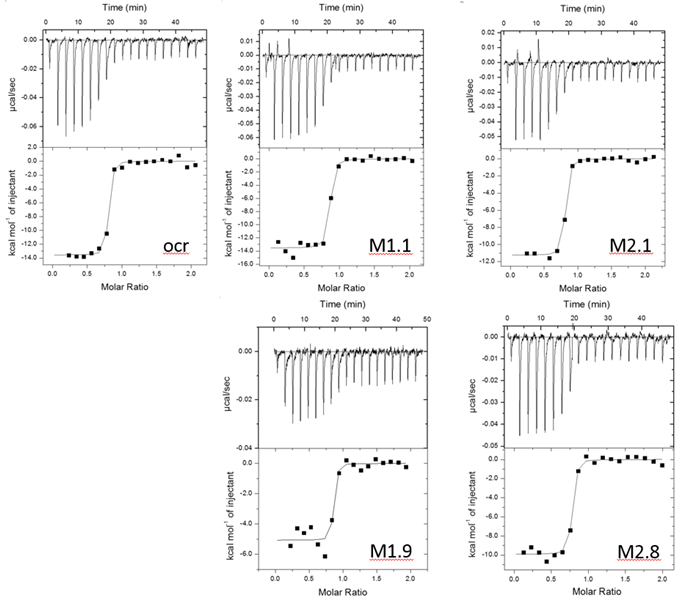


| Protein | Stoichiometry, N | ΔH (kcal/mol) | K_d_ (nM) | ΔS (cal/mol/Deg) |
| --- | --- | --- | --- | --- |
| Ocr | 0.76+/-0.01 | -13.5+/-0.2 | 0.54+/-0.26 | -5.45 |
| M2.8 | 0.74+/-0.26 | -9.9+/-0.2 | 0.49+/-0.25 | 6.58 |
| M2.1 | 0.77+/-0.003 | -11.3+/-0.1 | 0.40+/-0.12 | 1.63 |
| M1.1 | 0.80+/-0.01 | -13.5+/-0.3 | 0.34+/-0.22 | -6.34 |
| M1.9 | 0.82+/-0.02 | -5.7+/-0.2 | 0.75+/-0.01 | 23.6 |

Figure S7

Ordered in decreasing total charge in each subtype of variants identified in this study.

Wild type ocr is given as the first sequence in each section with secondary structure elements and the three regions corresponding to regions of the DNA target being mimicked are given with residues highlighted in green, orange and purple.

Active

HELIX A LOOP 1 HELIX B HELIX C

Structure ------hhhhhhhhhhhhhhhhhh---------hhhhhhhhhhh----hhhhhhhhh---

Regions MAMSNMTYNNVFDHAYEMLKENIRYDDIRDTDDLHDAIHMAADNAVPHYYADIFSVMASE

Wt_Ocr MAMSNMTYNNVFDHAYEMLKENIRYDDIRDTDDLHDAIHMAADNAVPHYYADIFSVMASE

Mut4 MAMSNMTYNNVFDHAYEMLKENIRYDDIRDTDDLHDAIHMAADNAVPHYYADIFSVMASE

Ocr109 MAMSNMTYNNVFDHAYEMLKENIRYDDIRDTDDLHDAIHMAADNAVPHYYADIFSVMASE

Mut1 MAMSNMTYNNVFNHAYQMLKQNIRYNNIRDTDDLHDAIHMAADNAVPHYYADIFSVMASE

Mut2 MAMSNMTYNNVFDHAYEMLKENIRYNNIRNTNNLHNAIHMAADNAVPHYYADIFSVMASE

Ocr99 MAMSNMTYNNVFDHAYEMLKENIRYDDIRDTDDLHDAIHMAADNAVPHYYADIFSVMASE

Mut7 MAMSNMTYNNVFDHAYEMLKENIRYNNIRNTNNLHNAIHMAADNAVPHYYADIFSVMASE

2.8 MAMSNMTYNNVFNHAYQMLKENIRYDDIRDTDDLHDAIHMAADNAVPHYYADIFSVMASE

2.1 MAMSNMTYNNVFNHAYQMLKENIRYNDIRDTNNLHNAIHMAADNAVPHYYADIFSVMASE

1.1 MAMSNMTYNNVFNHAYQMLKENIRYDNIRDTNDLHDAIHMAADNAVPHYYADIFSVMASE

1.9 MAMSNMTYNNVFNHAYQMLKENIRYNDIRDTDDLHNAIHMAADNAVPHYYADIFSVMASE

************:***:***:****::**:*::**:************************

LOOP 2 HELIX D TAIL

Structure ------------------hhhhhhhhhhhhhhhhhhhhhhhhhhhhh----------

Regions GIDLEFEDSGLMPDTKDVIRILQARIYEQLTIDLWEDAEDLLNEYLEEVEEYEEDEE

Wt_Ocr GIDLEFEDSGLMPDTKDVIRILQARIYEQLTIDLWEDAEDLLNEYLEEVEEYEEDEE

Mut4 GIDLEFEDSGLMPDTKDVIRILQARIYEQLTINLWQNAQNLLNEYLEEVEEYEEDEE

Ocr109 GIDLEFEDSGLMPDTKDVIRILQARIYEQLTIDLWEDAEDLLNEYLEEV--------

Mut1 GIDLEFEDSGLMPDTKDVIRILQARIYEQLTIDLWEDAEDLLNEYLEEVEEYEEDEE

Mut2 GIDLEFEDSGLMPDTKDVIRILQARIYEQLTIDLWEDAEDLLNEYLEEVEEYEEDEE

Ocr99 GIDLEFEDSGLMPDTKDVIRILQARIYEQLTIDLWEDAE------------------

Mut7 GIDLEFEDSGLMPDTKDVIRILQARIYEQLTINLWQNAQNLLNEYLEEVEEYEEDEE

2.8 GIDLQFQDSGLMPDTKDVIRILQARIYEQLTINLWENAQNLLNQYLQQVQEYQQNQQ

2.1 GIDLEFEDSGLMPDTKDVIRILQARIYEQLTIDLWENAQNLLNQYLQQVQEYQQNQQ

1.1 GIDLQFEDSGLMPNTKDVIRILQARIYEQLTIDLWQDAQNLLNQYLQQVQEYQQNQQ

1.9 GINLQFQDSGLMPDTKDVIRILQARIYEQLTIDLWQNAQNLLNQYLQQVQQYQENQQ

**:*:*:******:******************:**::*:

Figure S7 cont.

Inactive

HELIX A LOOP 1 HELIX B HELIX C

Structure ------hhhhhhhhhhhhhhhhhh---------hhhhhhhhhhh----hhhhhhhhh---

Regions MAMSNMTYNNVFDHAYEMLKENIRYDDIRDTDDLHDAIHMAADNAVPHYYADIFSVMASE

Wt_Ocr MAMSNMTYNNVFDHAYEMLKENIRYDDIRDTDDLHDAIHMAADNAVPHYYADIFSVMASE

Mut13 MAMSNMTYNNVFDHAYEMLKENIRYDDIRDTDDLHDAIHMAADNAVPHYYADIFSVMASQ

Mut11 MAMSNMTYNNVFDHAYEMLKENIRYNNIRNTNNLHNAIHMAADNAVPHYYADIFSVMASQ

Mut10 MAMSNMTYNNVFDHAYEMLKENIRYNNIRNTNNLHNAIHMAADNAVPHYYADIFSVMASQ

DG12 MAMSNMTYNNVFNHAYQMLKENIRYNDIRDTNNLHNAIHMAADNAVPHYYADIFSVMASQ

S8 MAMSNMTYNNVFNHAYEMLKENIRYNDIRDTNNLHNAIHMAADNAVPHYYADIFSVMASQ

S9 MAMSNMTYNNVFNHAYEMLKENIRYNDIRDTNNLHNAIHMAADNAVPHYYADIFSVMASQ

DG3 MAMSNMTYNNVFNHAYQMLKENIRYDDIRDTNNLHNAIHMAADNAVPHYYADIFSVMASE

MM6 MAMSNMTYNNVFNHAYQMLKENIRYDDIRDTNNLHNAIHMAANNAVPHYYANIFSVMASE

C2 MAMSNMTYNNVFNHAYQMLKENIRYNDIRDTNNLHNAIHMAANNAVPHYYADIFSVMASE

G8 MAMSNMTYNNVFNHAYQMLKQNIRYNDIRDTNNLHDAIHMAANNAVPHYYADIFSVMASQ

DG10 MAMSNMTYNNVFNHAYEMLKENIRYNDIRDTDDLHNAIHMAANNAVPHYYADIFSVMASE

B12 MAMSNMTYNNVFNHAYEMLKENIRYNDIRDTNNLHNAIHMAANNAVPHYYANIFSVMASE

H3 MAMSNMTYNNVFDHAYQMLKQNIRYNDIRNTNNLHDAIHMAANNAVPHYYANIFSVMASQ

G7 MAMSNMTYNNVFNHAYQMLKQNIRYNDIRDTNNLHNAIHMAADNAVPHYYANIFSVMASE

113.2 MAMSNMTYNNVFNHAYQMLKENIRYNNIRNTDNLHNAIHMAANNAVPHYYANIFSVMASE

3.2 MAMSNMTYNNVFNHAYQMLKENIRYDNIRDTNNLHNAIHMAANNAVPHYYANIFSVMASE

21+11 MAMSNMTYNNVFNHAYQMLKQNIRYNDIRNTNNLHNAIHMAANNAVPHYYANIFSVMASE

ED3 MAMSNMTYNNVFDHAYQMLKENIRYNNIRNTDNLHNAIHMAANNAVPHYYADIFSVMASQ

E5 MAMSNMTYNNVFDHAYQMLKQNIRYNDIRNTNNLHNAIHMAANNAVPHYYANIFSVMASQ

E12 MAMSNMTYNNVFNHAYEMLKENIRYDNIRNTNDLHNAIHMAANNAVPHYYANIFSVMASQ

A10 MAMSNMTYNNVFNHAYQMLKENIRYNNIRNTDNLHNAIHMAANNAVPHYYADIFSVMASQ

A2 MAMSNMTYNNVFNHAYQMLKENIRYNNIRNTDNLHNAIHMAANNAVPHYYADIFSVMASQ

A8 MAMSNMTYNNVFNHAYQMLKENIRYNNIRNTDNLHNAIHMAANNAVPHYYADIFSVMASQ

3.29 MAMSNMTYNNVFNHAYQMLKENIRYNDIRDTDNLHNAIHMAADNAVPHYYADIFSVMASE

D10 MAMSNMTYNNVFDHAYQMLKQNIRYNNIRNTNNLHDAIHMAANNAVPHYYANIFSVMASQ

ED10 MAMSNMTYNNVFNHAYQMLKQNIRYNNIRNTNNLHDAIHMAANNAVPHYYANIFSVMASQ

ED4 MAMSNMTYNNVFNHAYQMLKENIRYNNIRNTNNLHNAIHMAANNAVPHYYADIFSVMASQ

A11 MAMSNMTYNNVFDHAYQMLKQNIRYNDIRNTNNLHDAIHMAANNAVPHYYANIFSVMASQ

G2=2 MAMSNMTYNNVFNHAYQMLKQNIRYNNIRDTNNLHNAIHMAANNAVPHYYANIFSVMASQ

E2 MAMSNMTYNNVFNHAYQMLKQNIRYNNIRDTNNLHNAIHMAANNAVPHYYANIFSVMASQ

ED9 MAMSNMTYNNVFNHAYEMLKQNIRYNNIRDTNNLHNAIHMAANNAVPHYYANIFSVMASQ

2.5 MAMSNMTYNNVFNHAYQMLKENIRYNNIRNTNNLHDAIHMAANNAVPHYYANIFSVMASQ

D7 MAMSNMTYNNVFNHAYEMLKQNIRYNNIRNTNNLHNAIHMAANNAVPHYYANIFSVMASQ

H7 MAMSNMTYNNVFNHAYQMLKENIRYNNIRNTDNLHNAIHMAANNAVPHYYANIFSVMASQ

************:***:***:****::**:*::**:******:********:*******:

LOOP 2 HELIX D TAIL

Structure ------------------hhhhhhhhhhhhhhhhhhhhhhhhhhhhh----------

Regions GIDLEFEDSGLMPDTKDVIRILQARIYEQLTIDLWEDAEDLLNEYLEEVEEYEEDEE

Wt_Ocr GIDLEFEDSGLMPDTKDVIRILQARIYEQLTIDLWEDAEDLLNEYLEEVEEYEEDEE

Mut13 GINLQFQNSGLMPDTKDVIRILQARIYEQLTINLWQNAQNLLNEYLEEVEEYEEDEE

Mut11 GINLQFQNSGLMPDTKDVIRILQARIYEQLTIDLWEDAEDLLNEYLEEVEEYEEDEE

Mut10 GINLQFQNSGLMPDTKDVIRILQARIYEQLTINLWQNAQNLLNEYLEEVEEYEEDEE

DG12 GIDLEFQNSGLMPDTKDVIRILQARIYEQLTIDLWENAQNLLNQYLQQVQEYQQNQQ

S8 GIDLEFENSGLMPDTKDVIRILQARIYQQLTINLWQNAENLLNQYLQQVQEYQQNQQ

S9 GIDLEFENSGLMPDTKDVIRILQARIYQQLTINLWQNAENLLNQYLQQVQEYQQNQQ

DG3 GIDLQFENSGLMPDTKDVIRILQARIYQQLTINLWENAQNLLNQYLQQVQEYQQNQQ

MM6 GIDLQFQNSGLMPDTKDVIRILQARIYQQLTINLWENAEDLLNQYLQQVQQYQENEQ

C2 GIDLQFQNSGLMPNTKDVIRILQARIYQQLTIDLWEDAQNLLNQYLEQVQQYQENQE

G8 GINLQFQNSGLMPDTKDVIRILQARIYQQLTINLWENAEDLLNQYLQQVQQYEQNQE

DG10 GINLEFQNSGLMPNTKDVIRILQARIYQQLTINLWQNAQDLLNQYLQQVQQYQQNQQ

B12 GIDLQFQNSGLMPDTKDVIRILQARIYQQLTINLWENAQNLLNQYLQQVQEYQQNQQ

H3 GIDLQFQNSGLMPNTKDVIRILQARIYQQLTINLWQDAQNLLNQYLEQVQQYEQNQE

G7 GIDLQFQNSGLMPDTKDVIRILQARIYQQLTINLWQDAENLLNQYLQQVQQYQQNQQ

113.2 GIDLQFQNSGLMPDTKDVIRILQARIYQQLTINLWQNAQNLLNQYLQQVEQYQENQQ

3.2 GIDLQFQNSGLMPDTKDVIRILQARIYQQLTIDLWQNAQNLLNQYLQQVQQYQQNQQ

21+11 GIDLQFQNSGLMPDTKDVIRILQARIYQQLTINLWQDAEDLLNQYLQQVQQYQQNQQ

ED3 GINLQFENSGLMPNTKNVIRILQARIYQQLTINLWQNAENLLNQYLEQVQQYQENQQ

E5 GIDLQFQNSGLMPDTKNVIRILQARIYQQLTINLWENAQNLLNEYLQQVQEYQQNEQ

E12 GINLQFQDSGLMPNTKNVIRILQARIYQQLTINLWQDAQNLLNQYLEQVQQYEQNQQ

A10 GINLQFQNSGLMPNTKDVIRILQARIYQQLTINLWQDAQNLLNQYLQQVQQYEQNQE

A2 GINLQFQNSGLMPNTKDVIRILQARIYQQLTINLWQDAQNLLNQYLQQVQQYEQNQE

A8 GINLQFQNSGLMPNTKDVIRILQARIYQQLTINLWQDAQNLLNQYLQQVQQYEQNQE

3.29 GINLQFQNSGLMPNTKNVIRILQARIYQQLTINLWQNAQNLLNQYLQQVQQYQQNQQ

D10 GIDLQFQNSGLMPDTKNVIRILQARIYQQLTINLWENAQNLLNQYLQQVQEYQQNEQ

ED10 GIDLQFQNSGLMPDTKNVIRILQARIYQQLTINLWENAQNLLNQYLQQVQEYQQNEQ

ED4 GINLQFQNSGLMPNTKNVIRILQARIYEQLTINLWQNAENLLNQYLQEVQQYQENQQ

A11 GIDLQFQNSGLMPDTKNVIRILQARIYQQLTINLWQNAQNLLNQYLQQVQEYQQNQQ

G2=2 GINLQFQNSGLMPNTKDVIRILQARIYQQLTINLWQDAEDLLNQYLQQVQQYEQNQQ

E2 GINLQFQNSGLMPNTKDVIRILQARIYQQLTINLWQDAQNLLNQYLQQVQQYEQNQE

ED9 GINLQFQNSGLMPNTKDVIRILQARIYQQLTINLWQDAQNLLNQYLQQVQQYEQNQQ

2.5 GINLQFQNSGLMPDTKNVIRILQARIYQQLTINLWENAQNLLNQYLQQVQEYQQNQQ

D7 GINLQFQNSGLMPNTKDVIRILQARIYQQLTINLWQDAQNLLNQYLQQVQQYQQNQQ

H7 GINLQFQNSGLMPNTKNVIRILQARIYQQLTINLWQNAENLLNQYLQQVQQYQQNQQ

**:*:*::*****:**:**********:****:**::*::***:**::*::*:::::

Figure S7 cont.

PA and PA*

HELIX A LOOP 1 HELIX B HELIX C

Structure ------hhhhhhhhhhhhhhhhhh---------hhhhhhhhhhh----hhhhhhhhh---

Regions MAMSNMTYNNVFDHAYEMLKENIRYDDIRDTDDLHDAIHMAADNAVPHYYADIFSVMASE

Wt_Ocr MAMSNMTYNNVFDHAYEMLKENIRYDDIRDTDDLHDAIHMAADNAVPHYYADIFSVMASE

Mut12 MAMSNMTYNNVFDHAYEMLKENIRYDDIRDTDDLHDAIHMAADNAVPHYYADIFSVMASQ

Mut3 MAMSNMTYNNVFDHAYEMLKENIRYDDIRDTDDLHDAIHMAADNAVPHYYADIFSVMASQ

Mut16 MAMSNMTYNNVFNHAYQMLKQNIRYNNIRDTDDLHDAIHMAADNAVPHYYADIFSVMASQ

Ocr/pocr MAMSNMTYNNVFDHAYEMLKENIRYDDIRDTDDLHDAIHMAADNAVPHYYADIFSVMASE

2.4 MAMSNMTYNNVFNHAYEMLKQNIRYNDIRDTNNLHNAIHMAANNAVPHYYADIFSVMASE

B4 MAMSNMTYNNVFNHAYQMLKENIRYDNIRDTNNLHNAIHMAADNAVPHYYADIFSVMASE

113.4 MAMSNMTYNNVFNHAYQMLKQNIRYDNIRDTNNLHDAIHMAANNAVPHYYANIFSVMASE

113.3 MAMSNMTYNNVFDHAYQMLKENIRYDNIRDTNNLHNAIHMAANNAVPHYYANIFSVMASE

21+9 MAMSNMTYNNVFNHAYQMLKQNIRYNDIRDTNNLHNAIHMAANNAVPHYYANIFSVMASE

12+7 MAMSNMTYNNVFNHAYQMLKQNIRYDNIRDTNNLHNAIHMAANNAVPHYYANIFSVMASE

12+5 MAMSNMTYNNVFNHAYQMLKQNIRYDNIRDTNNLHNAIHMAADNAVPHYYANIFSVMASE

ED6 MAMSNMTYNNVFNHAYQMLKENIRYNNIRNTNNLHNAIHMAANNAVPHYYADIFSVMASQ

ED5 MAMSNMTYNNVFDHAYQMLKQNIRYNDIRNTNNLHNAIHMAANNAVPHYYANIFSVMASQ

12+8 MAMSNMTYNNVFNHAYQMLKQNIRYNDIRNTNNLHNAIHMAANNAVPHYYADIFSVMASQ

************:***:***:****::**:*::**:******:********:*******:

LOOP 2 HELIX D TAIL

Structure ------------------hhhhhhhhhhhhhhhhhhhhhhhhhhhhh----------

Regions GIDLEFEDSGLMPDTKDVIRILQARIYEQLTIDLWEDAEDLLNEYLEEVEEYEEDEE

Wt_Ocr GIDLEFEDSGLMPDTKDVIRILQARIYEQLTIDLWEDAEDLLNEYLEEVEEYEEDEE

Mut12 GINLQFQNSGLMPDTKDVIRILQARIYEQLTIDLWEDAEDLLNEYLEEVEEYEEDEE

Mut3 GINLQFQNSGLMPCTKDVIRILQARIYEQLTIDLWEDAEDLLNEYLEEVEEYEEDEE

Mut16 GINLQFQNSGLMPDTKDVIRILQARIYEQLTIDLWEDAEDLLNEYLEEVEEYEEDEE

Ocr/pocr GIDLEFEDSGLMPDTKNVIRILQARIYQQLTINLWQNAQNLLNQYLQQVQQYQQNQQ

2.4 GIDLQFQNSGLMPDTKDVIRILQARIYQQLTINLWQNAEDLLNQYLQQVQQYEQNEE

B4 GIDLQFQNSGLMPDTKDVIRILQARIYQQLTINLWENAQNLLNQYLQQVQEYQQNQQ

113.4 GIDLQFQNSGLMPDTKDVIRILQARIYQQLTINLWQNAENLLNQYLQQVEQYQQNEQ

113.3 GIDLQFQNSGLMPNTKDVIRILQARIYQQLTINLWQDAQNLLNQYLQQVQQYEQNQE

21+9 GIDLQFQNSGLMPDTKDVIRILQARIYQQLTINLWQNAQDLLNEYLQQVQEYQQNQQ

12+7 GINLQFQNSGLMPDTKDVIRILQARIYQQLTINLWEDAQNLLNQYLEQVQQYEQNQQ

12+5 GINLQFQNSGLMPNTKDVIRILQARIYQQLTINLWQDAEDLLNQYLQQVQQYQQNQQ

ED6 GINLQFENSGLMPNTKNVIRILQARIYEQLTINLWQNAENLLNQYLQQVQQYQENQQ

ED5 GIDLQFQNSGLMPDTKNVIRILQARIYQQLTINLWQNAQNLLNQYLQQVQEYQQNEQ

12+8 GINLQFQNSGLMPDTKDVIRILQARIYQQLTINLWQNAQDLLNQYLQQVQQYEQNQQ

**:*:*::***** **:**********:****:**::*::***:**::*::*:::::

Figure S7 cont.

Toxic

HELIX A LOOP 1 HELIX B HELIX C

Structure ------hhhhhhhhhhhhhhhhhh---------hhhhhhhhhhh----hhhhhhhhh---

Regions MAMSNMTYNNVFDHAYEMLKENIRYDDIRDTDDLHDAIHMAADNAVPHYYADIFSVMASE

Wt_Ocr MAMSNMTYNNVFDHAYEMLKENIRYDDIRDTDDLHDAIHMAADNAVPHYYADIFSVMASE

3.11 MAMSNMTYNNVFNHAYQMLKENIRYDNIRDTNDLHDAIHMAADNAVPHYYADIFSVMASE

21+8 MAMSNMTYNNVFDHAYQMLKQNIRYNDIRDTNNLHNAIHMAANNAVPHYYANIFSVMASE

DG4 MAMSNMTYNNVFNHAYQMLKENIRYDDIRDTNNLHNAIHMAADNAVPHYYADIFSVMASE

21+10 MAMSNMTYNNVFNHAYQMLKQNIRYNDIRDTNNLHNAIHMAANNAVPHYYADIFSVMASE

21+6 MAMSNMTYNNVFNHAYQMLKQNIRYDDIRDTNNLHNAIHMAANNAVPHYYANIFSVMASE

B7 MAMSNMTYNNVFNHAYQMLKENIRYDNIRDTNNLHNAIHMAADNAVPHYYANIFSVMASE

G2=7 MAMSNMTYNNVFNHAYQMLKQNIRYDNIRDTNNLHNAIHMAANNAVPHYYADIFSVMASE

21+1 MAMSNMTYNNVFNHAYQMLKQNIRYNDIRDTNNLHNAIHMAANNAVPHYYANIFSVMASE

2.1 MAMSNMTYNNVFNHAYQMLKQNIRYNDIRDTNNLHNAIHMAANNAVPHYYANIFSVMASE

MM2 MAMSNMTYNNVFNHAYQMLKQNIRYDNIRDTNNLHNAIHMAANNAVPHYYANIFSVMASQ

G1=8 MAMSNMTYNNVFNHAYQMLKQNIRYNNIRDTNNLHNAIHMAANNAVPHYYANIFSVMASQ

G2=8 MAMSNMTYNNVFNHAYQMLKENIRYNNIRNTNNLHNAIHMAANNAVPHYYADIFSVMASQ

ED5* MAMSNMTYNNVFNHAYQMLKENIRYNNIRNTNNLHNAIHMAANNAVPHYYANIFSVMASQ

************:*******:****::**:**:**:******:********:*******:

LOOP 2 HELIX D TAIL

Structure ------------------hhhhhhhhhhhhhhhhhhhhhhhhhhhhh----------

Regions GIDLEFEDSGLMPDTKDVIRILQARIYEQLTIDLWEDAEDLLNEYLEEVEEYEEDEE

Wt_Ocr GIDLEFEDSGLMPDTKDVIRILQARIYEQLTIDLWEDAEDLLNEYLEEVEEYEEDEE

3.11 GIDLQFEDSGLMPNTKDVIRILQARIYQQLTIDLWQDAQNLLNQYLQQVQEYQQNQQ

21+8 GIDLQFQNSGLMPDTKDVIRILQARIYQQLTINLWEDAEDLLNQYLQQVQQYEQNEQ

DG4 GIDLQFQNSGLMPDTKDVIRILQARIYQQLTINLWENAQNLLNQYLQQVQEYQQNQQ

21+10 GINLQFQNSGLMPDTKDVIRILQARIYQQLTINLWQDAQDLLNQYLQQVEQYQQDQE

21+6 GIDLQFQDSGLMPDTKDVIRILQARIYQQLTINLWQNAQNLLNQYLQQVEQYQQDQE

B7 GIDLQFQNSGLMPNTKNVIRILQARIYEQLTIDLWENAQNLLNQYLQQVQEYQQNQQ

G2=7 GIDLQFQDSGLMPNTKDVIRILQARIYQQLTINLWQNAENLLNQYLQQVEQYQQNQQ

21+1 GINLQFQNSGLMPNTKDVIRILQARIYQQLTINLWQNAEDLLNQYLEQVQQYQENEQ

2.1 GIDLQFQNSGLMPDTKDVIRILQARIYQQLTINLWQNAQNLLNQYLEQVQQYQENQQ

MM2 GINLQFQNSGLMPDTKDVIRILQARIYQQLTINLWQNAEDLLNQYLQQVQEYQQNQQ

G1=8 GINLQFENSGLMPNTKNVIRILQARIYEQLTINLWQNAENLLNQYLQQVQQYQQNQQ

G2=8 GINLQFQNSGLMPNTKNVIRILQARIYQQLTINLWQNAQNLLNQYLQQVEQYQQDQQ

ED5* GINLQFQNSGLMPNTKNVIRILQARIYQQLTINLWQNAENLLNQYLEQVQQYQENQQ

**:***::*****:**:**********:****:**::*::******:**::*:::::

Figure S8

Homologues to ocr identified by a BLASTP search of non-redundant protein sequences. Multiple sequences from phage T7 have been removed. The secondary structures and DNA-mimicking regions have been added to the ocr sequence.

Search sequence:

>Wt_Ocr MAMSNMTYNNVFDHAYEMLKENIRYDDIRDTDDLHDAIHMAADNAVPHYYADIFSVMASEGIDLEFEDSGLMPDTKDVIRILQARIYEQLTIDLWEDAEDLLNEYLEEVEEYEEDEE

Results:

Accession Description Links

NP_041954.1 hypothetical protein T7p01 [Enterobacteria phage T7]

AFK13391.1 protein 0.3 [Yersinia phage YpP-Y]

NP_848263.1 protein 0.3 [Yersinia phage phiA1122]

AFK13392.1 protein 0.3 [Yersinia phage YpP-R]

YP_009152459.1 protein kinase [Escherichia phage CICC 80001]

YP_009204325.1 protein kinase [Escherichia phage P694]

YP_002003935.1 gp0.3 [Enterobacteria phage 13a]

YP_009187268.1 Ocr [Yersinia phage vB_YenP_AP10]

YP_009205652.1 hypothetical protein RU52_00001 [Citrobacter phage phiCFP-1]

AKQ06760.1 hypothetical protein phiYe-F10_00001 [Yersinia phage phiYe-F10]

NP_853565.1 gp5 [Enterobacteria phage SP6]

YP_007500981.1 hypothetical protein Phi78p06 [Enterobacteria phage UAB_Phi78]

WP_048901937.1 hypothetical protein [Yersinia pseudotuberculosis]

YP_006987769.1 hypothetical protein ACG-C91_0007 [Enterobacteria phage vB_EcoP_ACG-C91]

YP_009007137.1 predicted antirestriction protein [Citrobacter phage CR44b]

YP_002003736.1 gp0.3 [Enterobacteria phage EcoDS1]

YP_424972.1 putative ocr protein [Enterobacteria phage K1E]

YP_338090.1 antirestriction protein [Enterobacteria phage K1F]

YP_009044250.1 hypothetical protein PE3_002 [Escherichia phage PE3-1]

YP_654101.1 hypothetical protein EPKV1_gp03 [Enterobacteria phage K1-5]

ALT58456.1 protein kinase [Klebsiella phage vB_KpnP_IME205]

YP_009004159.1 predicted antirestriction protein [Citrobacter phage CR8]

YP_006990205.1 hypothetical protein [Stenotrophomonas phage IME15]

YP_004678723.1 ocr protein [Enterobacteria phage K30]

YP_009190955.1 protein kinase [Klebsiella phage vB_Kp1]

HELIX A LOOP 1 HELIX B HELIX C LOOP 2

Structure ------hhhhhhhhhhhhhhhhhh---------hhhhhhhhhhh----hhhhhhhhh-------------------

Regions MAMSNMTYNNVFDHAYEMLKENIRYDDIRDTDDLHDAIHMAADNAVPHYYADIFSVMASEGIDLEFEDSGLMPDTK

Wt_Ocr MAMSNMTYNNVFDHAYEMLKENIRYDDIRDTDDLHDAIHMAADNAVPHYYADIFSVMASEGIDLEFEDSGLMPDTK

NP_041954 1 MAMSNMTYNNVFDHAYEMLKENIRYDDIRDTDDLHDAIHMAADNAVPHYYADIFSVMASEGIDLEFEDSGLMPDTK 76

AFK13391 1 MAMSNMTYKNVFDHAYEMLKENIRYDDIRDTDDLHDAIHMAADSAIPHYYSDIFSVMASEGIDLEFEDSGLMPDTK 76

NP_848263 1 MAMSNMTYKNVFDHAYEMLKENIRYDDIRDTDDLHDAIHMAADSAIPHYYSDIFSVMASEGIDLEFEDSGLMPDTK 76

AFK13392 1 MAMSNMTYKNVFDHAYEMLKENIRYDDIRDTDDLHDAIHMAADSAIPHYYSDIFSVMASEGIDLEFEDSGLMPDTK 76

YP_009152459 1 MAMSNMTYNNVFDHAYEMLKENTRYDDIRDTDDLHDAVHMAADSAVPHYYSDVFSVMASEGIDLEFEDSGLMPDTK 76

YP_009204325 1 MAMSNMTYNNVFDHAYEMLKENIRYDDIRDTDDLHDAVYMAAEIAVPHYYSDVFSVMASEGIDLEFEDSGLMPDTK 76

YP_002003935 1 MAMSNMTYNNVFDHAYEMLKENIRYDDIRDTDDLHDAIHEAADNAVPHYYSDIFSVMASEGIYHEFEDSGLMPDTK 76

YP_009187268 1 MAMSNMTYSNVFDHAYEMLNENIRYDDIRDTDGLGDAIHEAADSAVPHYYSDIFSVMASEGIDLEFEDSGLMPDTK 76

YP_009205652 1 [13]MAMSNMTYSNVFAHAYEMLNENIRYDDIRDTDGLGDAIHEAADSAVPHYYSDIFSVMASDGIDLEFEDSGLMPDTK 89

AKQ06760 1 MAMSNMTYSNVFAHAYEMLNENIRYDDIRDTDGLGDALHEAADSAVPHYYSDIFSVMASDGIDPEFEDSGLMPDTK 76

NP_853565 1 MAMSNMTYSDVYNHAYGLLKEYIRYDDVRNEDDLSDKIHEAAGNAVPHWYADIFSVMASDGIDLEFDDSGLMPDTK 76

YP_007500981 1 MSYSNMTYANVYNHAYSLLDEYIRYEDVRDYDEATDKIHEAADNAVPVYYKDIFSVMASGGIDHEFEDSGLIPDTK 76

WP_048901937 1 MAMSNMTYSQVYNNAHAALVERIWNDAIDCIDNAHDAIHDVADSAVPIYYADIFSVMASEGIDHFFEDSGLMPDTK 76

YP_006987769 1 MAMSNMTYSQVYNTAHAALVERIWDDAIDCIDNAHDAIHEVADSTVPIYYADIFSVMASEGIDHFFEDSGLMPDTK 76

YP_009007137 1 MERNVFAYYELLASTVEAFNERIQYDEIAEGDDYHDALQEVVDGQVPHYYHEIFTVMAADGIDLEFEDSGLMPETK 76

YP_002003736 1 MERNANAYYDLLAATVEAFNERIQYDKIAEGDDYHDALHEVVDGQVPHYYHEIFTVMAADGIDHEFEDSGLMPDTK 76

YP_424972 1 MERNANAYYNLLAATVEAFNERIQYDEIREGDDYSDALHEVVDSNVPVYYSEIFTVMAADGIDIEFEDAGLIPDTK 76

YP_338090 1 MERNANAYYELLAATVELFNERIQYDEITEGDDYSDALHEVVDGQVPHYYHEIFTVMAADGIDHEFEDSGLMPETK 76

YP_009044250 1 MERNANAYYELLAATVEAFNERIQYDKIAEGDDYSDALHEVVDGNVPHYYHEIFTVMAADGIDHEFEDSGLMPETK 76

YP_654101 1 MERNANAYYNLLAATVEAFNERIQFDEIREGDDYSDALHEVVDSNVPVYYSEIFTVMAADGIDVDFEDAGLIPDTK 76

ALT58456 1 MERNANAYYDLVAATVKLFNERIQYDELTENDDWSDALHEVVDGQVPHYYSEIFMVMAADGIDFEFNDSGLIPDTK 76

YP_009004159 1 MERNADAYYKLVADAVKAFNERIQYDQIAEGDEYHDALHEVVDDYVPHYYSEIFTVMAADGIDHEFEDSGLMPESM 76

YP_006990205 1 MERNANAYYELLAATVEAFNERIQEDQLTEHHDYHDALHEVVDRMVPHYYWEIFTVMAADGIDVEFDDAGLMPDTK 76

YP_004678723 1 MERNANAYYELLAATVEAFNEHIQYDKLTEDDDWYDALHEVVDSHVPHYYHEIFTVMAADGIDHEFDDSDLIPDTK 76

YP_009190955 1 MERNANAYYDLLAATIELFNDRIQQDELTEDDDWSDALHEVVDGQVPHYYSEIFTVMAADGIDHEFDDSGLIPDTK 76

Figure S8 cont.

HELIX D TAIL

--hhhhhhhhhhhhhhhhhhhhhhhhhhhhh----------

DVIRILQARIYEQLTIDLWEDAEDLLNEYL EE VEEYEEDEE

Wt_Ocr DVIRILQARIYEQLTIDLWEDAEDLLNEYL EE VEEYEEDEE

NP_041954 77 DVIRILQARIYEQLTIDLWEDAEDLLNEYL EE[ 2]EYEED-E[1] 117

AFK13391 77 DVIRILQARIYEQLTIDLWEDAEDLLNEYL EE[110]DKQLD-A[4] 228

NP_848263 77 DVIRILQARIYEQLTIDLWEDAEDLLNEYL EE[ 22]DKQLD-A[4] 140

AFK13392 77 DVIRILQARIYEQLTIDLWEDAEDLLNEYL EE[ 5]DYEED-E[1] 120

YP_009152459 77 DVIRILQARIYEQLTIDLWEDTEDLLNEYL EE[ 5]EEE---- 116

YP_009204325 77 DVIRILQARIYEQLTIDLWEDAEDLLNEYL EE[ 5]EYEED-E[1] 120

YP_002003935 77 DVTRILQARIYEQLTIDLWEDAEDLLNEYL EE[ 2]EEEED-L 116

YP_009187268 77 DVTSILQARIYEQLTIDLWEKAEDLLNEYL EE[ 2]EEEEErE[4] 121

YP_009205652 90 DVTCILQARIYEQLTIDLWEKAEDLLNEYL EE[ 2]EEEEEvE[4] 134

AKQ06760 77 DVTCILQARIYEQLTIDLWEKAEDLLNEYL EE[ 2]EEEEEvE[4] 121

NP_853565 77 DVTYILQARIHEQLTIDLYGDAEDLLNEYL EE[ 1]EAEEDeE[3] 119

YP_007500981 77 DVTRILQARIYEQLTIDLYEVVADLLNEYL EE[ 1]EAEEDeE[2] 118

WP_048901937 77 DVTVVLQARIYEQLTIDLWERVQYMIVEYI NS[ 1]EYLEEeE[3] 119

YP_006987769 77 DVTVVLQARIYEQLTIDLRERVQDMIDAYI DS[ 1]EDSEEeE[2] 118

YP_009007137 77 DVTRILQARIYEALYNDVSNSSHIVWFEAE[4]ED[ 2]------- 114

YP_002003736 77 DVTRILQARIYEALYNDVSNSSDVVWFE--[2]ED[ 2]------- 110

YP_424972 77 DVTKILQARIYESLYNDVPNDSDVVWYE--[2]EE ------- 108

YP_338090 77 DVTRILQARIYEALYNDVSNSSEVVWFE--[2]EE[ 61]------- 169

YP_009044250 77 DVTHILQARIYEALYIDVSNSSDVVWFE--[2]EG[ 2]------- 110

YP_654101 77 DVTKILQARIYEALYNDVPNDSDVVWCE--[2]EE[ 2]------- 110

ALT58456 77 DVSRICQARIYEALYNDVSNDSGVIWFEE-[2]ED[ 3]------- 112

YP_009004159 77 DVTVILQARIYEALYNDVQNDCGLVWYE--[2]EE[ 3]------- 111

YP_006990205 77 DVTRILQARIYEALYNDVPSDSGIEWYE--[2]EE[ 65]------- 173

YP_004678723 77 DVSRICQARIYEALYNDILNDSRVIWCEQG[4]ED[ 4]------- 116

YP_009190955 77 DVSRICQARIYEALYNDVSNDSGIIWYEEP[4]ED[ 3]------- 115

Table S1

Sequences of the mutagenic oligonucleotides used in the modified ISOR procedure. The mutagenic oligonucleotides were divided into four separate pools to generate different sublibraries (1-4). In each case, the mutated codon is shown in bold typeface.

| **Oligonucleotide** | **Library** | **Sequence (5'-3')** |
| --- | --- | --- |
| N13Df | 1 | CAATAACGTTTTT**GAT**CACGCATATCAGA |
| N27Df | 1 | CATTCGCTACAAT**GAC**ATTCGTAATACCA |
| N36Df | 1 | CAACAACCTGCAC**GAC**GCCATCCACATGG |
| N63Df | 1 | TTCTCAGGGTATC**GAC**CTGCAGTTCCAGA |
| N74Df | 1 | CGGCCTGATGCCG**GAC**ACCAAAAACGTAA |
| Q96Ef | 1 | TATCAACCTGTGG**GAA**AACGCGCAGAACC |
| Q104Ef | 1 | GAACCTGCTGAAC**GAG**TATCTGCAACAGG |
| Q111Ef | 1 | GCAACAGGTGCAG**GAA**TATCAGCAGAACC |
| Q116Ef | 1 | ATATCAGCAGAAC**GAA**CAGTAAAAGCTTG |
| Q17Er | 2 | TCTGTTTCAGCAT**CTC**ATATGCGTGATTA |
| N30Df | 2 | CAATAACATTCGT**GAT**ACCAACAACCTGC |
| N43Df | 2 | CCACATGGCCGCG**GAC**AACGCAGTCCCAC |
| Q65Ef | 2 | GGGTATCAACCTG**GAG**TTCCAGAACTCCG |
| N77Df | 2 | GCCGAACACCAAA**GAC**GTAATCCGTATTC |
| N97Df | 2 | CAACCTGTGGCAA**GAC**GCGCAGAACCTGC |
| Q107Ef | 2 | GAACCAGTATCTG**GAA**CAGGTGCAGCAAT |
| Q113Ef | 2 | GGTGCAGCAATAT**GAG**CAGAACCAACAGT |
| Q117Ef | 2 | TCAGCAGAACCAA**GAG**TAAAAGCTTGACG |
| Q21Ef | 3 | TCAGATGCTGAAA**GAG**AACATTCGCTACA |
| N32Df | 3 | CATTCGTAATACC**GAC**AACCTGCACAACG |
| N52Df | 3 | ACACTACTACGCG**GAC**ATCTTCAGCGTTA |
| Q67Ef | 3 | CAACCTGCAGTTC**GAG**AACTCCGGCCTGA |
| Q88Ef | 3 | GGCTCGTATCTAC**GAG**CAGCTGACTATCA |
| Q99Ef | 3 | GTGGCAAAACGCG**GAG**AACCTCCTGAACC |
| Q108Ef | 3 | CCAGTATCTGCAA**GAG**GTGCAGCAATATC |
| Q114Ef | 3 | GCAGCAATATCAG**GAG**AACCAACAGTAAA |
| N26Dr | 4 | TATTACGAATGTT**ATC**GTAGCGAATGTTC |
| N33Df | 4 | TCGTAATACCAAC**GAC**CTGCACAACGCCA |
| Q60Ef | 4 | CGTTATGGCTTCT**GAG**GGTATCAACCTGC |
| N68Df | 4 | CCTGCAGTTCCAG**GAC**TCCGGCCTGATGC |
| N93Df | 4 | GCAGCTGACTATC**GAC**CTGTGGCAAAACG |
| N100Df | 4 | GCAAAACGCGCAG**GAC**CTGCTGAACCAGT |
| Q110Ef | 4 | TCTGCAACAGGTG**GAG**CAATATCAGCAGA |
| N115Df | 4 | GCAATATCAGCAG**GAC**CAACAGTAAAAGC |

Table S2

Sequences of the mutagenic oligonucleotides used in the third round of the modified ISOR procedure. In each case, the mutated codons are shown in bold typeface. Cluster 1 refers to the Helix A and Loop 1 (aa 26-36) region. Cluster 2 refers to the Loop 2 (aa 60-68) region. Cluster 3 refers to the Helix D and Tail region (aa 96-100, aa 107-111 and aa 113-117).

| **Oligonucleotide** | **Residue Target Sites** | **Cluster** | **Sequence (5’-3’)** |
| --- | --- | --- | --- |
| N26_27f | 26,27 | 1 | CATTCGCTAC**GATGAC**ATTCGTAATACC |
| N26_30f | 26,30 | 1 | CATTCGCTAC**GAT**AACATTCGT**GAT**ACC |
| N27_30f | 27,30 | 1 | CATTCGCTACAAT**GAC**ATTCGT**GAT**ACC |
| N32-36r | 32,33,36 | 1 | GATGGC**GTY**GTGCAG**GTYGTY**GGT**ATC**ACG |
| N60-63f | 60,63 | 2 | GCGTTATGGCTTCT**GAG**GGTATC**GAC**CTG |
| Q65-68r | 65,67,68 | 2 | CGGA**GTYCTS**GAA**CTS**CAG**GTC**GATACC |
| N96-100f | 96,97,99,100 | 3 | CTGTGG**SAARAC**GCG**SAGRAC**CTGCTGAAC |
| N107-111f | 107,108,110,111 | 3 | TATCTG**SAASAG**GTG**SAGSAA**TAT |
| N113-117r | 113,114,115,116,117 | 3 | CGCCAAGCTTTTA**CTSTTSGTYCTSCTS**ATA |

Table S3

Excel spreadsheet of mutants picked at random from libraries 1, 2 and 3.
